# Chemical Vapor Deposition Mediated Phase Engineering for 2D Transition Metal Dichalcogenides: Strategies and Applications

**DOI:** 10.1002/smsc.202100047

**Published:** 2021-10-19

**Authors:** Karla Hernandez Ruiz, Ziqian Wang, Matteo Ciprian, Meifang Zhu, Rong Tu, Lianmeng Zhang, Wei Luo, Yuchi Fan, Wan Jiang

**Affiliations:** ^1^ State Key Laboratory for Modification of Chemical Fibers and Polymer Materials Institute of Functional Materials College of Materials Science and Engineering, Donghua University Shanghai 201620 China; ^2^ Department of Materials Science and Engineering Johns Hopkins University Baltimore MD 21218 USA; ^3^ State Key Laboratory of Advanced Technology for Materials Synthesis and Processing Wuhan University of Technology Wuhan 430070 China

**Keywords:** 2D transition metal dichalcogenides, chemical vapor deposition, phase-selective growth

## Abstract

2D transition metal dichalcogenides (TMDs) are characterized by the presence of multiple crystal structures or phases, even at the ultrathin limit. Controlling phase transformations, namely, phase engineering, of 2D TMDs is crucial for realizing high‐performance 2D devices by combining phases with distinct physical and chemical properties. As a powerful approach for large‐scale production of high‐quality 2D TMDs, chemical vapor deposition (CVD) offers unique advantages in phase engineering due to its highly controllable synthesis processes. Starting with an introduction of the crystal structures and phase transformations of 2D TMDs, this review summarizes the recent developments in CVD‐mediated phase engineering strategies of TMDs, including control of temperature, precursors, catalysis, atmosphere, composition, and strain during the deposition process. Moreover, the representative applications of CVD‐based phase‐engineered TMDs in the field of transistors, photodetectors, photovoltaic cells, and catalysis are overviewed. Finally, the challenges, expectations of CVD‐based phase engineering, and future development of this versatile technique are discussed.

## Graphical abstract

A detailed review of the recent development in phase engineering of 2D transition metal dichalcogenides (2D TMDs) by chemical vapor deposition (CVD) technique is presented, in which the CVD‐oriented controlling strategies for various 2D TMDs and representative applications of phase‐selective grown materials in high‐performance devices are highlighted.

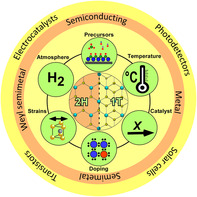

## 1 Introduction

Since the first time that scientists managed to isolate a single sheet of graphene in 2004,^[^ ^1^ ^]^ 2D materials have become one of the most attractive research fields in materials science for their unprecedented properties.^[^ 1, 2 ^]^ In the past decade, the 2D material family has expanded to hundreds of members, including elemental materials such as germanene, silicene, borophene, and phosphorene and compounds such as 2D transition metal carbides/nitrides (MXenes) and 2D transition metal dichalcogenides (TMDs), just to name a few.^[^ ^3^ ^]^ In particular, 2D TMDs include members with a wide range of properties, providing an important platform for diverse electronic and photonic applications including transistors, photodetectors, memories, energy devices, and photovoltaic and electroluminescent devices by themselves or in combination with other 2D materials.^[^ ^4^ ^]^ Moreover, the discovery of polymorphism, the presence of multiple phases leading to very different physical and chemical properties, further expands the multifunctionality of 2D TMDs. For example, in a monolayer MoS_2_ field‐effect transistor (FET), when the areas in contact with the metal electrodes are transformed from semiconducting 2H to metallic 1T phase, the contact resistance is reduced by a factor of five compared with directly forming contacts on 2H.^[^ ^5^ ^]^ Likewise, in a MoS_2_ optoelectronic device, due to the reduction of Schottky barrier height at the 1T MoS_2_‐metal contacts, the photoresponsivity has been improved by an order of magnitude.^[^ ^6^ ^]^ It has also been demonstrated that the metastable 1T phase MoS_2_ exhibits significantly improved catalytic activity for the hydrogen evolution reaction (HER) compared with the stable 2H phase, and 1T MoS_2_ shows a much higher specific capacitance than its 2H counterpart.^[^ 7, 8, 9 ^]^ Therefore, manipulating different phases of the 2D TMDs for different purposes, or the so‐called phase engineering, is of great importance not only for fundamental research but also for practical applications.

The phase conversion of 2D MoS_2_ was first achieved by alkali ion intercalation and has now been developed as a common technique for inducing phase transformations in many TMDs.^[^ ^10^ ^]^ The transformation from the ambient stable 2H phase to the metastable 1T phase is triggered by the electron transfer from the *s* orbital of alkali metal to the *d* orbital of transition metal.^[^ ^11^ ^]^ The intercalation can be realized by two approaches, namely, the electrochemical intercalation and the solution‐based intercalation, respectively.^[^ ^12^ ^]^ However, the use of alkali metal or lithium‐containing compounds is obviously neither cost‐effective nor environmentally friendly for scalable production of phase‐engineered TMDs. Furthermore, the obtained film usually contains flakes with inhomogeneous size and thickness, which severely limits the possibility of application in electronic devices.

**Figure 1:**
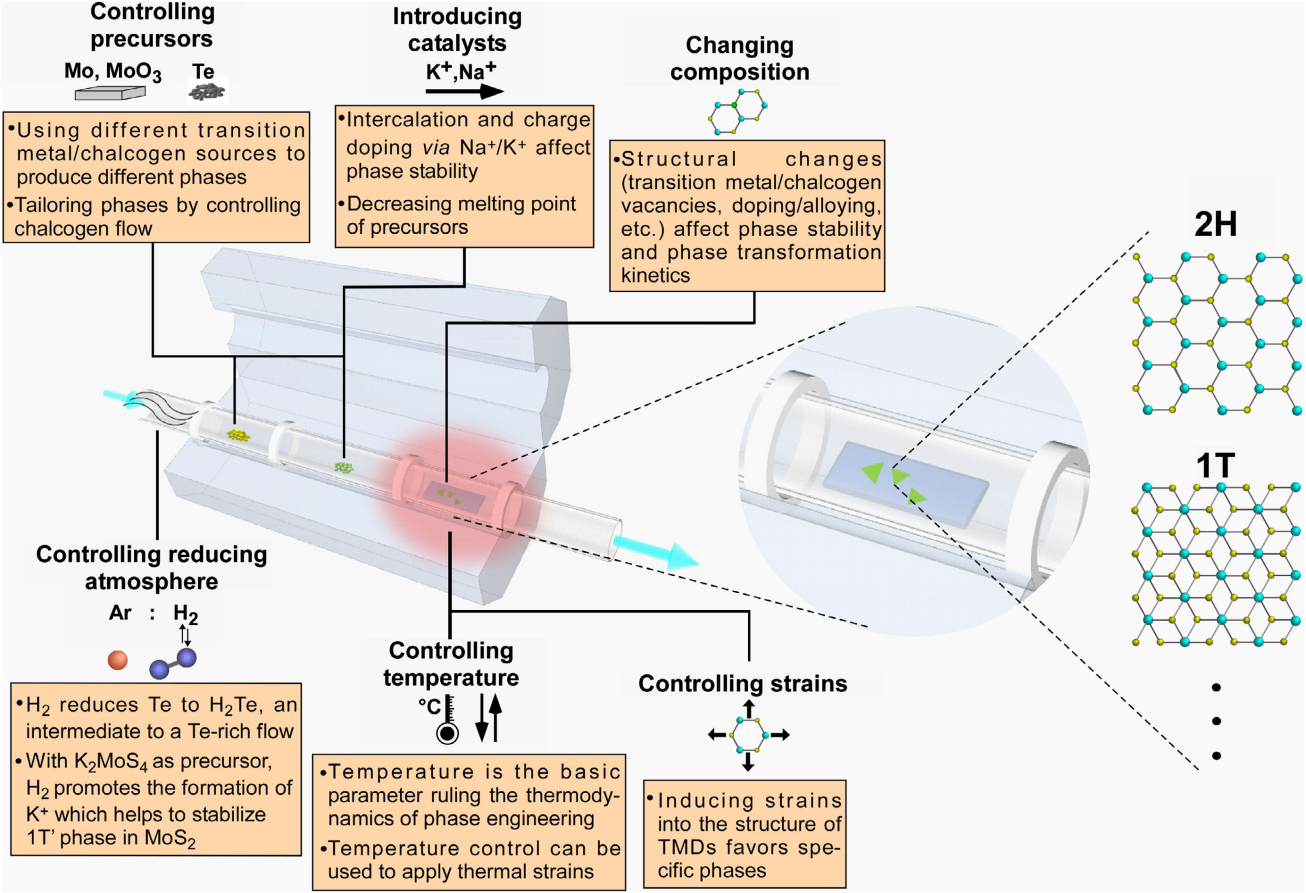
Scheme of the characteristic CVD setup for 2D TMDs growth and the approaches to CVD‐mediated phase engineering.

In contrast to the top‐down strategy described earlier, chemical vapor deposition (CVD) offers a bottom‐up solution for preparing high‐quality 2D TMDs in various phases. Typically, a CVD process produces a thin film of the target material through the adsorption and reaction of volatile precursors. Taking the most common CVD growth of monolayer MoS_2_ as an example, the precursor MoO_3_ is partially reduced by S vapor (to form highly volatile MoO_3–*x*_), and then S and MoO_3–*x*_ are transported to the substrate and react to nucleate and grow into the typical triangle‐shaped monolayer MoS_2_ flakes. Further growth and coalescence of flakes will continue under a longer deposition time. Various 2D TMDs have been grown successfully by CVD on different substrates such as SiO_2_,^[^ 13, 14 ^]^ sapphire,^[^ ^15^ ^]^ mica,^[^ ^16^ ^]^ glass,^[^ ^17^ ^]^ glassy carbon,^[^ ^18^ ^]^ and Au foil,^[^ ^19^ ^]^ to name a few. CVD is a powerful technique that provides films or flakes with excellent crystallinity and precisely controlled thickness and size by designing synthesis conditions. Furthermore, large‐area uniform deposition is a major advantage of the CVD method, which can be combined with recent patterning techniques for scalable fabrication.^[^ ^20^ ^]^ Intriguingly, recent reports have demonstrated that the phase of TMDs can also be tailored by adjusting CVD conditions, which adds new flexibility to the CVD‐based synthesis techniques.^[^ 21, 22, 23 ^]^ Various strategies for selectively controlling the phase of TMDs in CVD processes have been studied (**Figure** **1**), and these approaches contribute significantly to the determination of the final structure of the material. Therefore, it is of important guiding significance to review and summarize the recent advances in CVD techniques for 2D TMD phase engineering, as well as the underlying principles and mechanisms, for promoting the application of these techniques and phase‐engineered 2D TMDs in a variety of fields. Although previous comprehensive reviews have introduced in a broad sense the polymorphism of 2D TMD, their phase engineering, and device applications ,^[^ ^24^ ^]^ a technology‐oriented summary focusing on the CVD techniques in TMD phase engineering, which is greatly useful for scalable phase‐selective production of TMD materials, has been lacking.

In this review, we first introduce the phase structures of 2D TMDs in Section 2, and then briefly introduce the widely used methods for phase characterization and identification in the literature in Section 3. As the most important part, Section 4 summarizes the approaches for CVD‐based phase engineering to provide a valuable guidance for producing 2D TMDs with desirable phases and thus functions and properties. Finally, in Section 5, we overview the notable applications of CVD‐based phase‐engineered TMDs and outlook the future of this technology.

## 2 Crystal Structure, Properties and Phase Transformations of 2D TMDs

**Figure 2:**
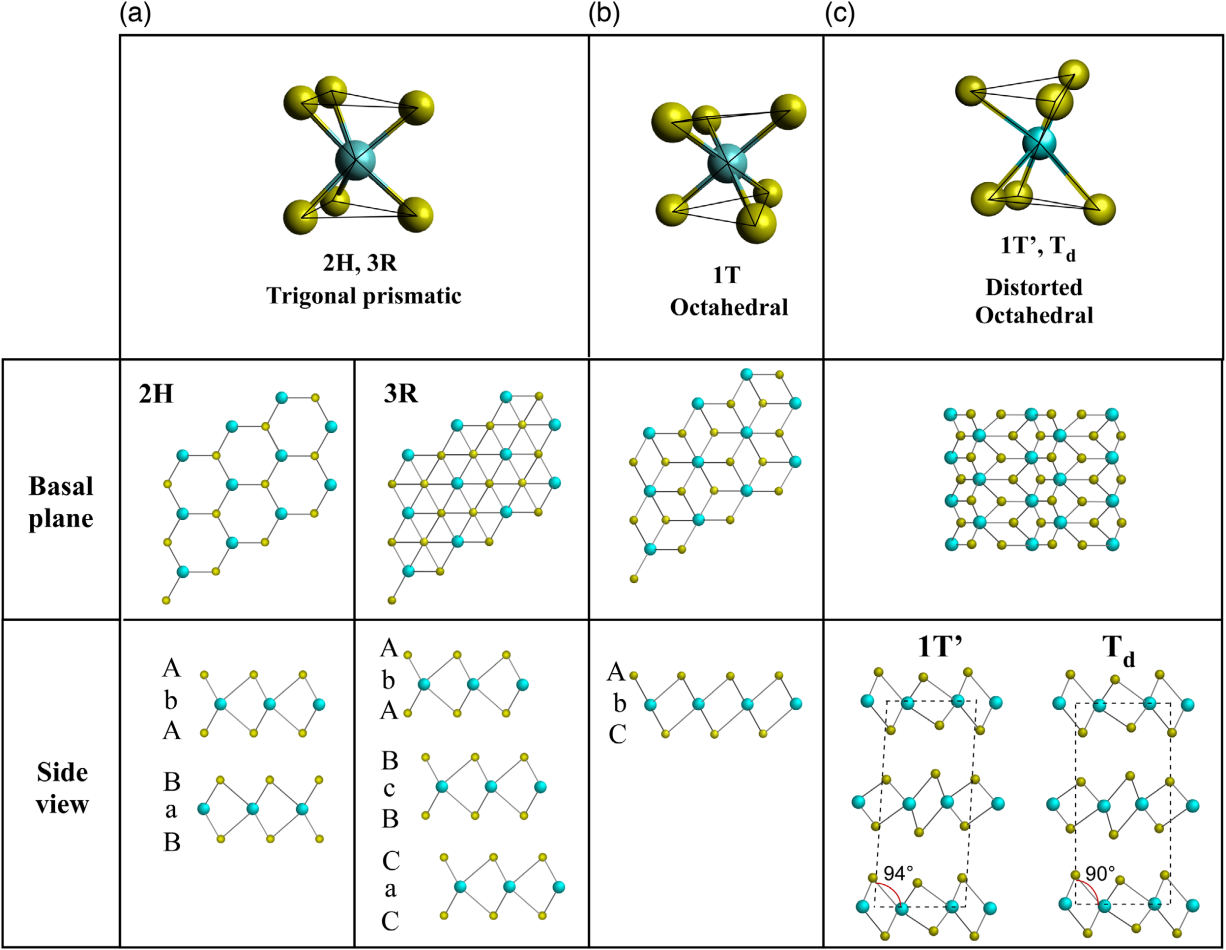
Schematic illustration of the metal coordination, basal plane, and side view of a) 2H and 3R, b) 1T, and c) 1T′ and T_d_ phases of TMDs. The basal plane and side view of every phase are also exhibited.

The chemical formula of the TMD family is MX_2_, where M is the transition metal and X is the chalcogen. Bulk TMDs have a layered crystal structure for all phases, with each layer having one layer of transition metal atoms sandwiched between two layers of chalcogen atoms. Despite the simple chemical formula, TMDs are unique for their polymorphism depending on the arrangement of the atoms in the atomic layers. The crystal structure of TMDs can be commonly classified into three different phases: 2H, 3R, and 1T (including derivatives such as 1T′, T_d_, etc.). The numbers (1, 2, and 3) denote the number of layers in a single stacking unit and the letters correspond to hexagonal (H), rhombohedral (R), and trigonal (T) lattice symmetries, respectively.^[^ ^25^ ^]^ Among these crystal structures, 2H and 1T phases have been extensively studied, especially over the past decade.^[^ ^26^ ^]^ In the 2H phase, the transition metal M is surrounded by six chalcogen X atoms with trigonal prismatic coordination, whereas in the 1T phase the transition metal coordination is octahedral. Therefore, the 2H phase and the 1T phase differ by the AbA and AbC stacking order within the layer, respectively, where A and C represent the positions of chalcogen atomic planes and b represents the transition metal atomic plane (**Figure** **2**). In other words, 1T shows a different sequence from 2H, with one of the 2H chalcogen planes spanning the hollow center of the 2H structural arrangement.^[^ ^27^ ^]^ In comparison, the 3R phase has a different layer stacking sequence compared with the 2H phase, while the stacking order within each layer remains the same.

Different structures of TMDs with the same chemical composition have drastically different physical properties, e.g., 2H MoS_2_ are semiconducting while 1T MoS_2_ exhibits metallic behavior. First proposed by Fernando Wypych and Robert Schöllhorn in 1991, the 1T phase of group 6 TMDs can be further stabilized by the formation of superlattice structures as a consequence of Peierls distortion, giving rise to multiple variants including the distorted octahedral coordinated 1T′ phase, orthorhombic T_d_ phase, and others under specific temperature and pressure.^[^ 28, 29, 30 ^]^ These variants of 1T phase are known to be metallic or semimetallic.

**Figure 3:**
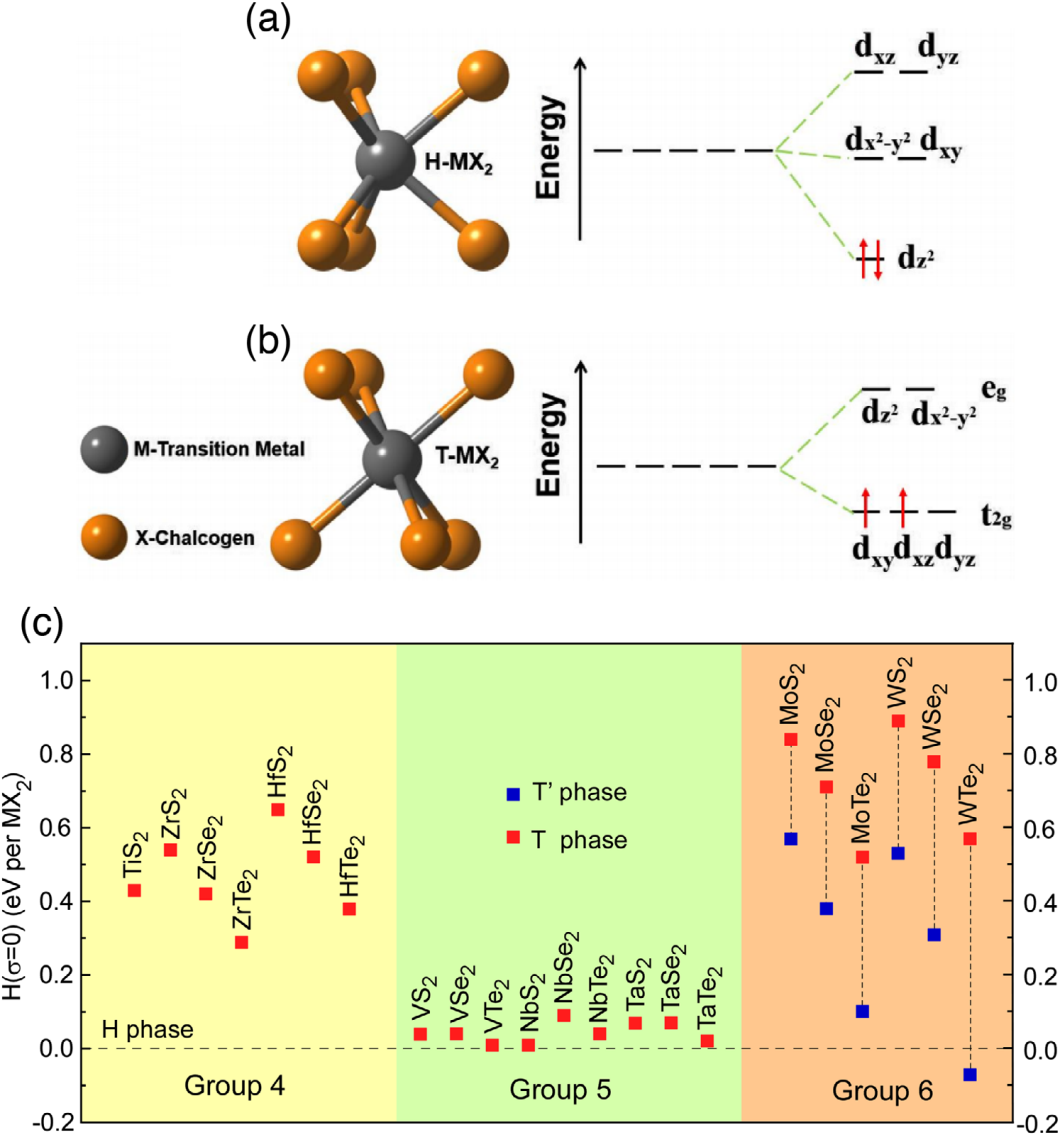
a) Splitting of the 3*d* orbital energy levels of a trigonal prismatic (H phase) and b) octahedral (T phase) crystal field. Electron filling of group 6 TMDs is shown as an example. c) Stress‐free (zero stress, *σ*) energy difference among phases of monolayer TMDs. Because *σ* = 0, Energy U is equivalent to the enthalpy H. TMDs as classified by its transition metal group. a,b) Reproduced with permission.^[^ ^31^ ^]^ Copyright 2017, IOP Publishing Ltd. c) Illustration of the data presented in Ref. [31].

The thermodynamic selectivity of the TMD phases can be roughly explained by the electron counting in the transition metal *d* orbitals.^[^ ^29^ ^]^ The octahedral coordination in the 1T phase leads to *d*_*z*_^2^,_*x*_^2^_‐*y*_^2^ (*e*_g_) (twofold) and *d*_*xy*,*yz*,*zx*_ (*t*_2g_) (threefold) crystal field splitting, as shown in **Figure** **3**a. In contrast, for the 2H phase, the *d* orbitals of the transition metal are divided into three energy levels, which are *d*_*z*_^2^ (*a*_1_), *d*_*xy*,*x*_^2^_‐*y*_^2^ (*e*) (twofold), and *d*_*xz*,*yz*_ (*e*’) (twofold) under the trigonal prismatic crystal field (Figure 3b). The number of electrons in the transition metal *d* orbital varies from 0 (*d* ^*0*^, group 4 TMDs) to +6 (*d* ^*6*^, group 10 TMDs) because the oxidation state of the transition metal in the TMD structure is equal to +4. As typically the *d*_*xy*,*yz*,*zx*_ levels of the 1T structure locate between the *d*_*z*_^2^ and *d*_*xy*,*x*_^2^_‐*y*_^2^ levels of the 2H structure, increasing the number of *d* electrons to more than six tends to favor the 1T structure. This explanation of phase stability by electron counting can be demonstrated not only by comparing TMDs from different groups, but also by the phase transformation triggered by direct electron injection. In situ scanning transmission electron microscopy tracked the 2H to 1T structural phase transformation of MoS_2_ at various stages in the process, indicating that the phase stability of TMDs is affected by the density of electrons in the *d* orbitals.^[^ ^27^ ^]^

Although a wide variety of TMDs have been observed to host 2H and 1T structures, from the perspective of the energy difference of polymorphs, the difficulty of their phase engineering differs significantly. Figure 3c shows the stress‐free energy corresponding to the polymorphs of monolayer TMDs. Based on density functional theory (DFT) calculations, it has been determined that group 4 TMDs are more stable in the 1T phase.^[^ ^31^ ^]^ The distorted 1T phase, which is induced by commensurate charge density wave (CDW) instability, was also considered in the DFT analysis for groups 4 and 5 TMDs. The results revealed that even though the relaxation for the 1T′ phase is conducted, the final optimized structure will present the 1T phase, indicating the absence of CDW instability.^[^ ^31^ ^]^

Group 5 TMDs are more stable in the 2H polytype, but the energy difference between the 2H and 1T phase is as small as less than 0.1 eV, allowing the possibility of the coexistence of the two phases. For group 6 TMDs, the 2H phase is the most stable one except for the special case of WTe_2_ that exhibits a thermodynamically stable 1T′ phase under ambient conditions. Taking this group as an example, the phase engineering of telluride TMDs is generally easier than their sulfide counterparts due to the much smaller energy difference between 2H and 1T′ phases.^[^ 32, 33 ^]^ Finally, for groups 7–10 TMDs the 1T, 1T′, and T_d_ phases are typically stable.

**Figure 4:**
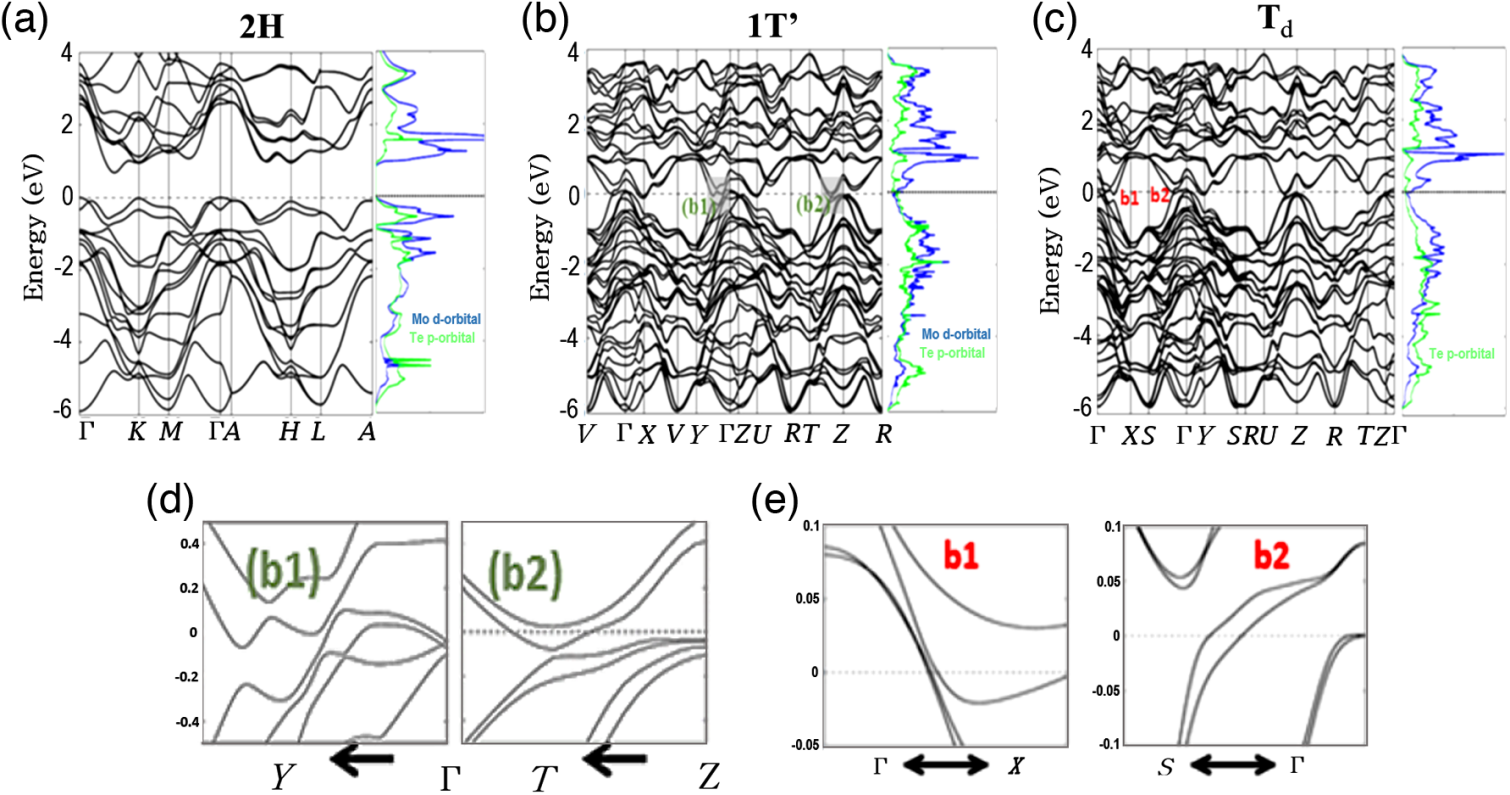
Band structures of MoTe_2_ polytypes. a–c) 2H, 1T′, and T_d_ phases. d,e) Enlarged images close to the Fermi level (*E*_F_) for (d) 1T′ and c) T_d_. a–e) Reproduced with permission.^[^ ^126^ ^]^ Copyright 2016, American Chemical Society.

The electronic characteristics of these TMDs are closely associated with their specific phases.^[^ ^34^ ^]^ A summary of the electronic properties of MX_2_ with M = Mo, W, Ta, Nb, V and X = S, Se, Te is shown in **Table** **1**. Some metallic TMDs display unique properties such as CDW and magnetism, but these are beyond the scope of this review and will not be discussed here. The 2H phase of TMDs such as MoX_2_ and WX_2_ (X = S, Se, and Te) exhibit semiconducting behavior, but the 2H phase can be metallic for certain TMDs such as NbX_2_ and TaX_2_ (X = S, Se, and Te). The 1T, 1T′, and T_d_ phases are generally metallic (except the semiconducting 1T′ ReS_2_, etc.) and have different band structures of special physical interest. Taking MoTe_2_ as an example, **Figure** **4** shows the differences in the band structures of 2H, 1T′, and Td phases. 2H MoTe_2_ is a semiconductor (Figure 4a) with a bandgap of ≈1.1 eV, while the 1T′ phase presents a metallic ground state (Figure 4b,d). In addition, the band structure of the Td phase (Figure 4c,e) shows that it has Weyl nodes near the Fermi‐level (*E*_F_) (set at 0 eV), resulting in the type‐II Weyl semimetal phase.^[^ ^35^ ^]^ In this review, unless specifically emphasized, we refer to the Weyl semimetal phase as well as other semimetal phases as metallic phases for simple description in the following discussion of phase engineering.

**Table 1:** Electronic and magnetic properties of different polytypes of 2D TMDs

Material	Phase	Properties	Ref.	Material	Phase	Properties	Ref.	Material	Phase	Properties	Ref.
**MoS**_**2**_	2H	Semiconducting		**WS**_**2**_	3R	Semiconducting		**TaSe**_**2**_	2H	Metallic	
		Bandgap of monolayer:	[128]			Superconducting at high	[129]			Superconducting	[130]
		1.88 eV and bulk: 1.23 eV				pressure				CDW	
	3R	Semiconducting	[131]		1T	Metallic	[132]		3R	Semiconducting	[133]
		Bandgap of 1.416 eV									
					1T′	Metallic	[134, 135]		1T	Metallic	[136]
	1T′	Metallic		**WSe**_**2**_	2H	Semiconducting				CDW	
		Superconducting	[127, 135]			Bandgap of monolayer:	[128, 137]				
		Monolayer: QSH insulator				1.67 eV and bulk: 1.21 eV		**NbS**_**2**_	2H	Metallic	[138]
										Superconducting	
	1T	Metallic	[8]		1T	Metallic	[139]				
**MoSe**_**2**_	2H	Semiconducting							3R	Semiconducting	[140]
		Bandgap of monolayer:	[128, 141]		1T′	Metallic					
		1.57 eV and bulk: 1.09 eV				Monolayer: QSH	[135, 142]		1T	Metallic	[143]
						Insulator					
	3R	Semiconducting	[144]					**NbSe**_**2**_	2H	Metallic	
				**WTe**_**2**_	2H	Semiconducting	[145]			Superconducting	[146]
	1T	Metallic	[147]			Monolayer: 1.07 eV				CDW	
	1T′	Metallic	[135, 148]		1T′	Semimetallic			1T	Mott insulator	[149]
		Monolayer: QSH insulator				Monolayer: QSH	[135, 150]			CDW	
**MoTe**_**2**_	2H	Semiconducting				Insulator					
		Bandgap of monolayer:	[151]					**NbTe**_**2**_	2H	Metallic	[152]
		1.1 eV and bulk: 1.0 eV			T_d_	Weyl semimetal					
						Induced	[153]		1T	Metallic	[154]
	1T	Metallic	[58]			superconductivity					
								**VS**_**2**_	2H	Semiconducting	
	1T′	Metallic		**TaS**_**2**_	2H	Metallic				Bulk bandgap: 0.5 eV	[155]
		Monolayer: QSH	[126, 135]			Superconducting	[156, 157]			Ferromagnetic	
		insulator				CDW					
	T_d_	Weyl semimetal	[158]						1T	Metallic	[159]
		Superconducting			3R	Metallic	[160]				
								**VSe**_**2**_	2H	Semiconducting	[161]
	3R	Semiconducting	[58]		1T	Metallic					
		Bandgap: ≈ 1 eV				Superconducting	[162]		1T	Metallic	[161, 163]
**WS**_**2**_	2H	Semiconducting				CDW				CDW	
		Bandgap of monolayer:	[128, 164]								
		2.03 eV and bulk:1.32 eV						**VTe**_**2**_	1T	Metallic	[165]
										CDW	

## 3 Characterization of Phase‐Engineered 2D TMDs

Reliable identification of different phases of 2D TMDs is an important prerequisite for the development of phase engineering techniques and phase‐related device applications. Typical material characterization methods include transmission electron microscopy (TEM), Raman (and photoluminescence) spectroscopy, and X‐ray photoemission spectroscopy (XPS). As each method has different advantages and limitations, a combination of different characterization methods is highly desirable for accurate phase identification.

### 3.1 TEM

**Figure 5:**
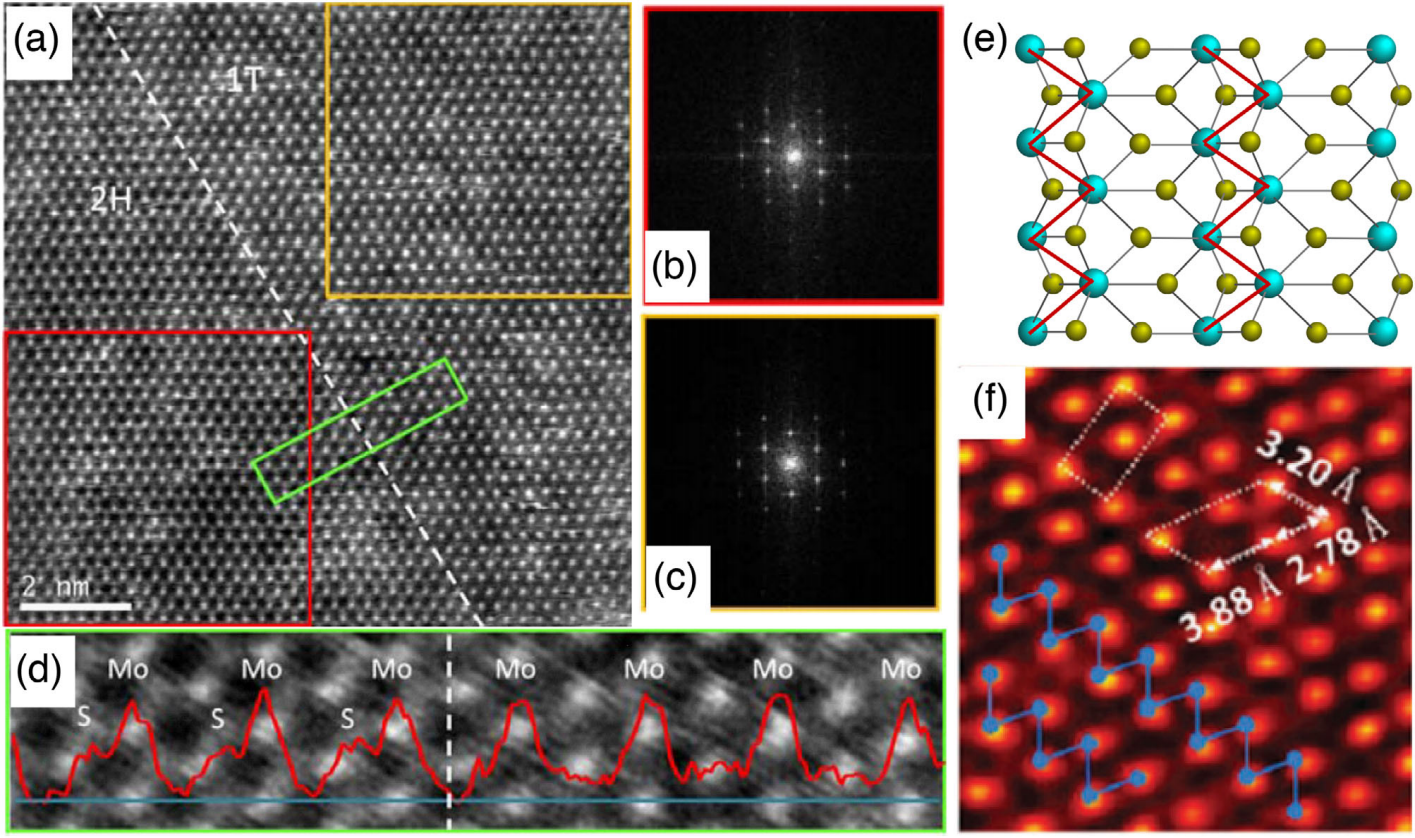
a) A STEM‐ADF image taken at the phase boundary (dashed line). The left and right domains present the 2H and 1T phases of MoS_2_, respectively. FFT patterns of b) 2H and c) 1T MoS_2_. d) The ADF intensity profile taken from the green area in (a). The intensity profile taken at the blue line is placed on the image which width corresponds to 3.9 nm. a–d) Reproduced with permission.^[^ ^36^ ^]^ Copyright 2012, American Chemical Society. e) Structure model and f) HAADF image of 1T′ phase MoS_2_, showing three different Mo—Mo distances with clear Mo—Mo zig—zag chains. e,f) Reproduced with permission.^[^ ^127^ ^]^ Copyright 2017, Royal Society of Chemistry.

TEM‐based techniques are powerful for directly observing the crystal structures of 2D TMDs. In the atomic‐resolution scanning TEM (STEM) mode with an annular dark‐field (ADF) or a high‐angle ADF (HAADF) detector, the atomic intensity is approximately proportional to a fixed power of the atomic number Z (known as Z‐contrast). By analyzing the arrangement and intensity of atomic columns, important information of the atomic structure and thus the phase of the 2D TMDs can be directly obtained. An exemplary STEM‐ADF image of a phase boundary of monolayer MoS_2_ is shown in **Figure** **5**a. Figure 5b,c shows the fast Fourier transform (FFT) patterns of semiconducting and metallic MoS_2_, respectively, revealing their different atomic structure. From the intensity profiles along the dashed line, the overlapping and staggered arrangements of S atoms clearly show the difference between 2H and 1T phases (Figure 5d). The intensity of the overlapping double S atoms in the phase is almost twice that of a staggering top or bottom single S atom in the 1T phase, and depending on imaging conditions and contamination level, a single S atom may not be clearly observable.^[^ ^36^ ^]^ The 1T′ phase of monolayer MoS_2_ can be identified by the presence of Mo zigzag chains (Figure 5e,f).

Despite the straightforward visualization of atomic structures by STEM, this technology is limited by the insufficient resolution in the electron beam direction which is often parallel to the *c*‐axis of 2D TMDs. Therefore, accurate identification of the 3D atomic structures by STEM remains elusive. The combination of STEM with selected area electron diffraction (SAED) in a TEM has been shown to resolve this issue under certain conditions.^[^ ^37^ ^]^ A more comprehensive summary of the STEM characterization of 2D TMDs can be found in the review work by Zhao et al.^[^ ^38^ ^]^

### 3.2 Raman Spectroscopy

**Figure 6:**
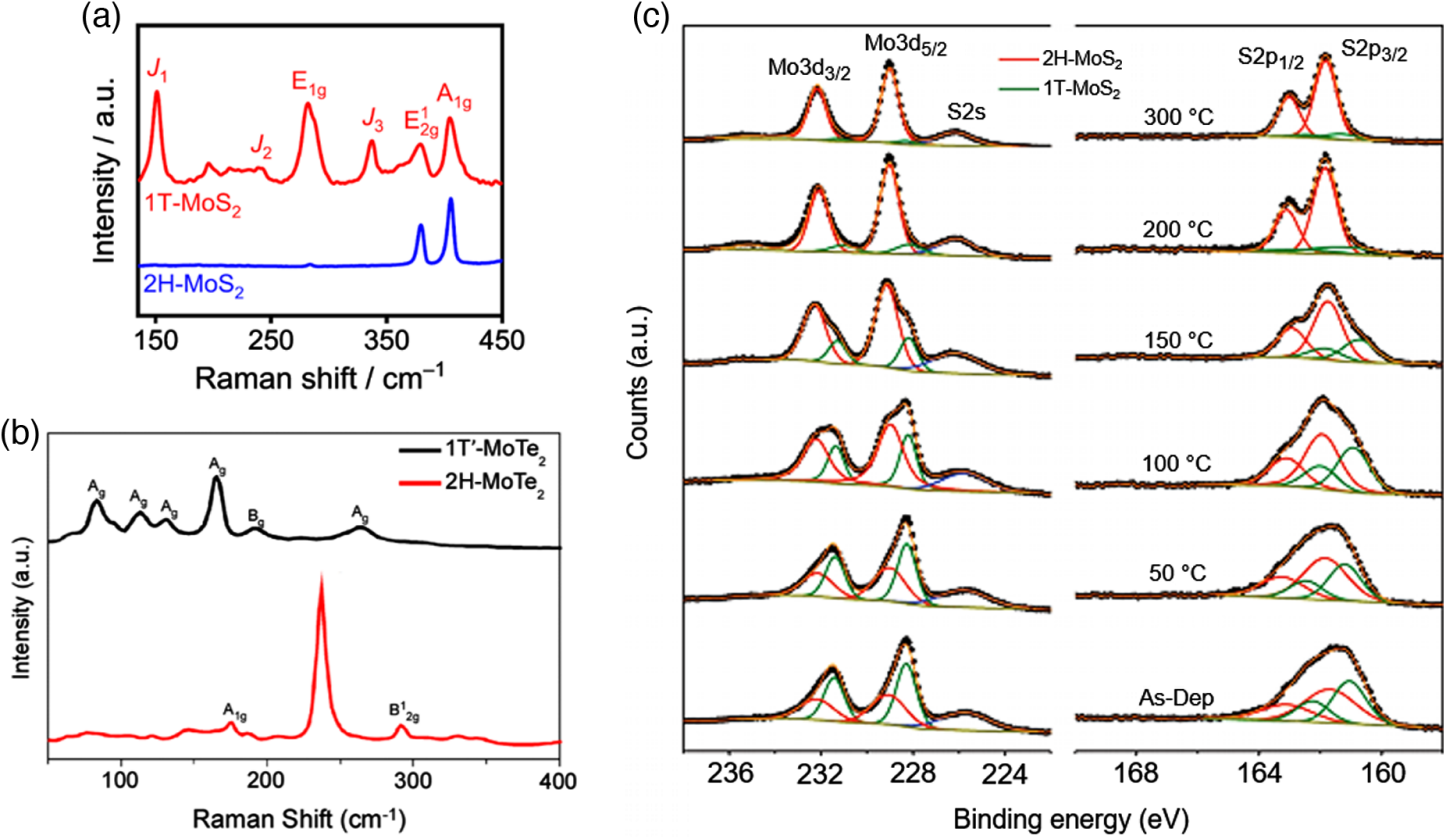
a) Raman spectra of 1T and 2H phases of MoS_2_ measured by a 532 nm laser. Reproduced with permission.^[^ ^41^ ^]^ Copyright 2020, National Academy of Sciences. b) Raman spectra of 2H and 1T′ phases of MoTe_2_ measured by a 532 nm laser. Reproduced under the terms of the CC‐BY 4.0 license.^[^ ^44^ ^]^ Copyright 2020, The Authors, published by Springer Nature. c) XPS analysis of Mo 3*d*, S 2*s*, and S 2*p* core levels of samples annealed at different temperatures. The deconvolution shows the contributions of 2H and 1T of MoS_2_ phases represented in red and green, respectively. Reproduced with permission.^[^ ^46^ ^]^ Copyright 2011, American Chemical Society.

Raman spectroscopy, which represents another approach to identifying TMD phases, can provide valuable information about the crystal symmetry, lattice vibrations, and thickness of the materials. By laser excitation in the visible region (typically 488, 514.5, 532, and 632.8 nm), the analysis provides spectral fingerprints that aids in identifying crystals by Raman scattering. The phases of TMDs can be determined by their Raman‐active phonon modes and symmetry selection rules. For instance, as shown in **Figure** **6**a, 2H MoS_2_ presents two characteristic modes at 379 cm^−1^ (E^1^_2g_ mode) and 405 cm^−1^ (A_1g_ mode), while 1T MoS_2_ exhibits additional peaks at 150, 239, and 324 cm^−1^ which are assigned to the J_1_, J_2_, and J_3_ vibrational modes, respectively.^[^ 39, 40, 41 ^]^ In the case of 1T′ MoS_2_, it presents peaks located at 152 cm^−1^ (A_2_ mode), 304 cm^−1^ (A_g_ mode), 326 cm^−1^ (A_1_ mode), and 401 cm^−1^ (A_1g_ mode).^[^ ^42^ ^]^ Because Raman spectroscopy is sensitive to the changes in symmetry, it is capable of distinguishing 1T phase derivatives, including 1T′ and T_d_, which cannot be identified using other spectroscopic techniques such as XPS. Polymorphs of MoTe_2_ have also been studied by Raman spectroscopy; 1T′ and 2H phases have been identified (Figure 6b).^[^ 43, 44 ^]^ The characteristic Raman peaks of 2H MoTe_2_ corresponding to the E^1^_2g_, A_1g_, and B^1^_2g_ modes can be found at 174, 234, and 289 cm^−1^, respectively. A reduced symmetry is exhibited by the 1T′ with peaks indexed as A_g_ modes at 80, 108, 125, 163, and 265 cm^−1^ and a B_g_ mode at 186 cm^−1^, respectively. More comprehensive reviews on the Raman characterization of 2D TMDs can be found in the works of Zhang et al.^[^ ^45^ ^]^

### 3.3 XPS

Different from TEM and Raman spectroscopy which only identify the phases qualitatively, XPS represents an effective characterization technique for quantitative analysis of different TMDs phases. Although it is difficult to use XPS for distinguishing the phases of similar electronic structures such as 1T, 1T′, and T_d_, the advantage of quantitative analysis of the 2H/1T(1T′) phase fraction by integrating the core level peaks from 2H and 1T phases makes XPS an essential characterization method for the phase engineering of 2D TMDs. For instance, Figure 6c shows the deconvoluted XPS spectra of Mo *3d*, S *2s*, and S *2p* core levels of MoS_2_.^[^ 46, 47 ^]^ The phase transition has been triggered by a simple heat treatment, at temperatures higher than 100 °C the 1T portion decreases and after 200 °C the 2H is the predominant phase in the material.

In addition to the characterization methods introduced earlier, other techniques have also shown their important role in identifying the TMD phases, such as scanning tunneling microscopy (STM), infrared spectroscopy, electrical transport measurements, and so on. But they will not be introduced in the present review because of their less popularity.

## 4 Approaches for 2D TMD Phase Engineering by CVD

As shown in Section 2, TMDs with different phases present unique properties, and a great number of recent studies have focused on the development of methodologies to produce TMDs with specific phases. The fact that the CVD method covers a broad range of materials and phases reveals its potential for phase engineering in combination with sample growth. **Table** **2** shows a summary of the CVD conditions for synthesizing various TMDs with different phases. In fact, a CVD process involves many controllable parameters, and these parameters may have different roles in influencing the phase transformation according to different mechanisms. This section overviews the techniques of controlling these parameters and their roles in phase engineering.

**Table 2:** CVD synthesis conditions for different 2D TMDs and their polytypes

Material	Phase	Precursor(s)/Promoters	Substrate	Synthesis conditions	Remarks	Ref.
**MoS**_**2**_	2H	MoO_3_ and S powder	SiO_2_/Si	Atmospheric pressure at 650 °C under N_2_ flow	Conventional CVD setup	[166]
	1T′	K_2_MoS_4_	Fluorophlogopite mica	Atmospheric pressure at 750 °C under Ar and H_2_ (10%) flow	The adsorbed K ions were removed by washing the 1T′ phase films with abundant water	[42]
	3R	MoO_3_ and S powder	SiO_2_/Si	750 °C under N_2_ and H_2_ (10%) flow	Reverse flow CVD setup, the direction of the gas flow can be reversed	[53]
**WS**_**2**_	2H	WO_3_ and S powder	SiO_2_/Si	750 °C under Ar flow	Two steps CVD heating program	[167]
	3R	WO_3_ and S powder	SiO_2_/Si	965 °C under Ar flow	Two steps CVD heating program	[168]
	1T	WO_3_ and S powder/NaCl and Fe_2_O_3_	Polished sapphire	Pressure of 0.1 Torr at 850 °C under Ar and H_2_ (7%) flow	Two steps CVD heating program. Fe_2_O_3_ is mixed with WO_3_	[78]
	1T′	K_2_WS_4_	Fluorophlogopite mica	Atmospheric pressure at 850 °C under Ar and H_2_ (12%) flow	The adsorbed K ions were removed by washing the 1T′ phase films with abundant water	[42]
**MoSe**_**2**_	2H	MoO_3_ and Se powder	SiO_2_/Si	Pressure of 20 Torr at 800 °C under H_2_ flow	Conventional CVD setup	[169]
**WSe**_**2**_	2H	WO_3_ and Se pellets	Au foil	Atmospheric pressure at 950 °C under Ar and H_2_ (1%) flow	Polished Au foil was annealed at 1095 °C for 10 h to reduce its roughness	[170]
	1T	W film grown on a sapphire substrate and Se powder	Sapphire substrate	Atmospheric pressure at 850 °C under Ar and H_2_ (24%) flow	Two ceramic boats containing Se powder were placed at two different zones at 40 and 850 °C	[171]
**MoTe**_**2**_	2H	MoO_3_ and Te powder	SiO_2_/Si	700 °C under Ar and H_2_ (57%) flow	Conventional CVD setup. Molecular sieves were added to absorb byproducts	[56]
	3R	MoCl_5_ and Te powder	Mica	360–520 °C under Ar and H_2_ (9%) flow	Conventional CVD setup	[58]
	1T	MoO_3_ and Te powder	SiO_2_/Si	650 °C under Ar and H_2_ (50%) flow	Two steps CVD heating program	[48]
	1T′	MoO_3_ and Te powder/ NaCl	SiO_2_/Si	650‐800 °C under Ar and H_2_ (33%) flow	NaCl and MoO_3_ were mixed and molecular sieves were also added.	[77]
	T_d_	MoCl_5_, MoO_3_ and Te powder	SiO_2_/Si	730 °C under Ar and H_2_ (16%) flow	Te powder was placed alone on a ceramic boat and Te+MoO_3_ + MoCl_5_ were mixed in another boat to lower their melting point	[172]
**WTe**_**2**_	1T′	Ammonium molybdate tetrahydrate and Te powder/NaCl	SiO_2_/Si	Atmospheric pressure at 800 °C under Ar and H_2_ (10%) flow	Ammonium molybdate tetrahydrate and NaCl were mixed with water and the solution was heated to remove the solvent	[173]
	T_d_	WO_*x*_ film grown on a glass substrate and Te powder	Glass	Atmospheric pressure at 700 °C under N_2_ and H_2_ (57%) flow	Conventional CVD setup	[174]
**TaS**_**2**_	2H	TaCl_5_ and S powder	Au foil	Atmospheric pressure and low pressure at 750 °C under Ar and H_2_ (9%) flow	Fast cooling by opening the furnace after the growth time is finished	[157]
	1T	Ta, Te and S powder/KCl	Sapphire	Pressure of 710 Torr at 850 °C under Ar atmosphere	Ta and Te powder are mixed and KCl is also added in the same ceramic boat	[79]
**TaSe**_**2**_	2H	TaCl_5_ and Se powder	Au foil	Atmospheric pressure at 930 °C under Ar and H_2_ (23%) flow	Fast cooling by opening the furnace after the growth time is finished	[175]
	3R	Ta, TaCl_5_ and Se powder	SiO_2_/Si	Atmospheric pressure at 800 °C under Ar and H_2_ (20%) flow	Ta and TaCl_5_ powder were mixed. After the reaction, the furnace was cooled down to 600 °C and then the top cover was opened	[176]
**NbS**_**2**_	2H	Nb_2_O_5_ and S powder/NaCl	SiO_2_/Si	Atmospheric pressure at 800 °C under Ar and H_2_ (6.3%) flow	Nb_2_O_5_ and NaCl were mixed. NaCl greatly decreased Nb_2_O_5_ melting point	[177]
	3R	Nb_2_Cl_5_ and S powder	SiO_2_/Si	Low pressure at 1000 °C under Ar and H_2_ (10%) flow	Two steps CVD heating program	[113]
	1T	NbCl_5_ with S(SiMe_3_)_2_ and Bu^t^SSBu^t^	Glass with a thick blocking layer of SiCO	Atmospheric pressure at 500–600 °C under Ar flow	The precursors were heated in individual canisters outside of the reactor and their vapor was carried inside by Ar gas	[178]
**NbSe**_**2**_	2H	NbO_*x*_ and Se powder/NaCl	SiO_2_/Si	Atmospheric pressure at 795 °C under Ar and H_2_ (17%) flow	By ignition of N powder at high temperature, NbO_*x*_ was obtained. NbO_*x*_ and NaCl were mixed in the same boat	[179]
**HfS**_**2**_	1T	HfCl_4_ and S powder	Sapphire	Pressure of 0.15 Torr at 1000 °C under Ar and H_2_ (33%) flow	Two steps CVD heating program	[180]
**ReS**_**2**_	Distorted 1T	Re, Te, and S powder	Fluorophlogopite mica	Atmospheric pressure at 700 °C under Ar flow	Te and Re powder were mixed. Te assisted to lower the melting point of Re	[181]
**ReSe**_**2**_	Distorted 1T	ReO_3_ and Se powder	Au foil	Atmospheric pressure at 750 °C under Ar and H_2_ (17%) flow	Au foil was annealed at 970 °C for 8 h to reduce its roughness	[182]

**Figure 7:**
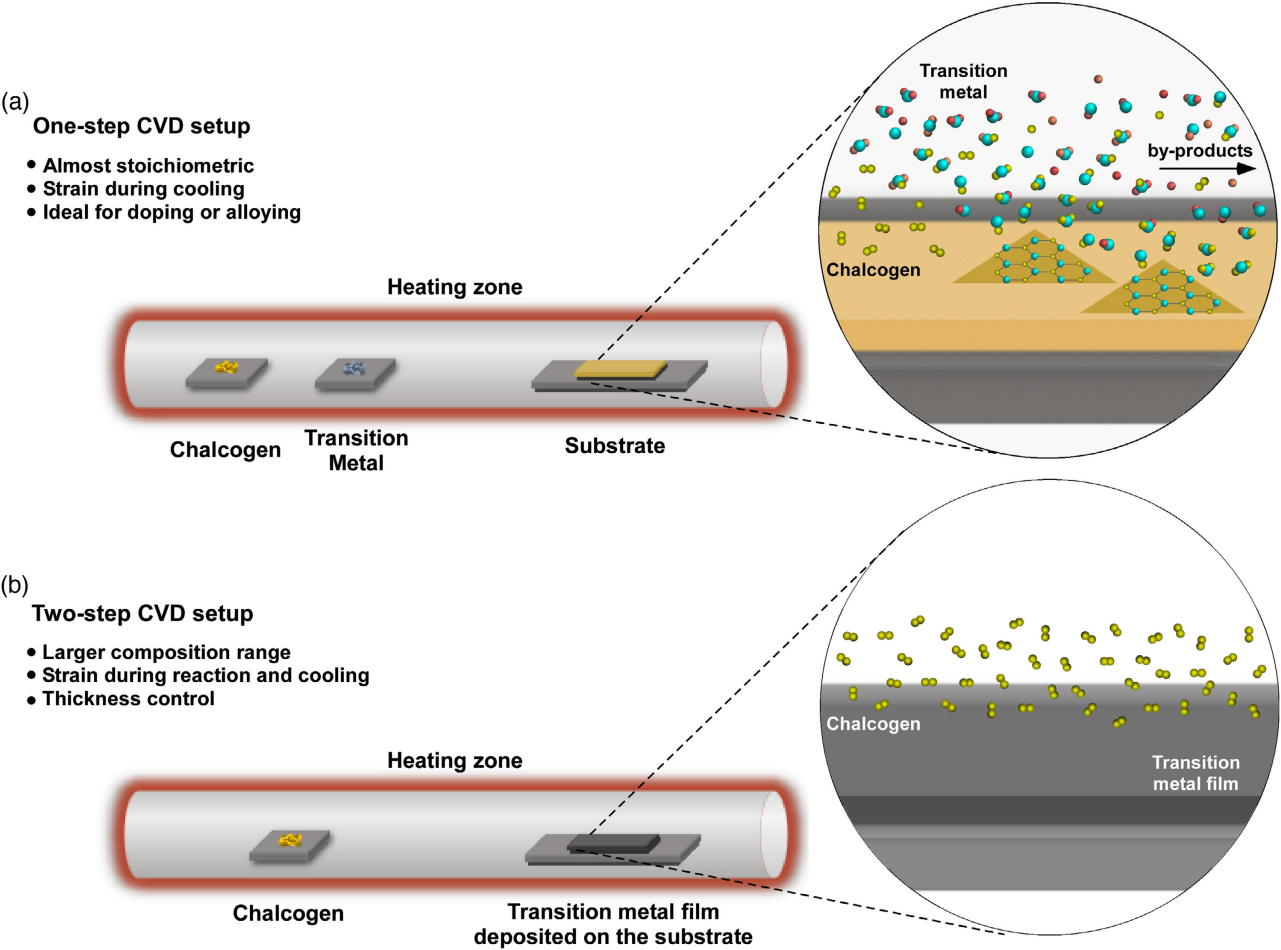
Schematic illustration of the a) one‐step and b) two‐step route for the deposition of 2D TMDs by CVD.

Previous studies on the CVD preparation of 2D TMDs can be broadly classified into two categories according to the process of chemical reactions. The first includes only a one‐step reaction (**Figure** **7**a), during which the transition metal and chalcogen precursors are vaporized and react to form TMD flakes or thin films. In contrast, the other type of CVD involves the deposition of a metal or metal oxide film on the substrate as the first step, followed by a sulfurization, selenization, or tellurization process of the predeposited film as the second step (Figure 7b). Typically, at an early stage of growth, the one‐step CVD produces 2D TMDs with triangular flakes, which can be extended to a continuous film by flake‐merging when the deposition time is sufficiently long while the direct production of a continuous film is common in the case of a two‐step CVD. This difference in deposition process leads to different ways to control the CVD parameters, including 1) temperature, 2) precursors, 3) catalysts, 4) reducing atmosphere, 5) composition and defects, and 6) strain, for the phase engineering of 2D TMDs, which will be discussed in detail as follows.

### 4.1 Controlling Temperature

**Figure 8:**
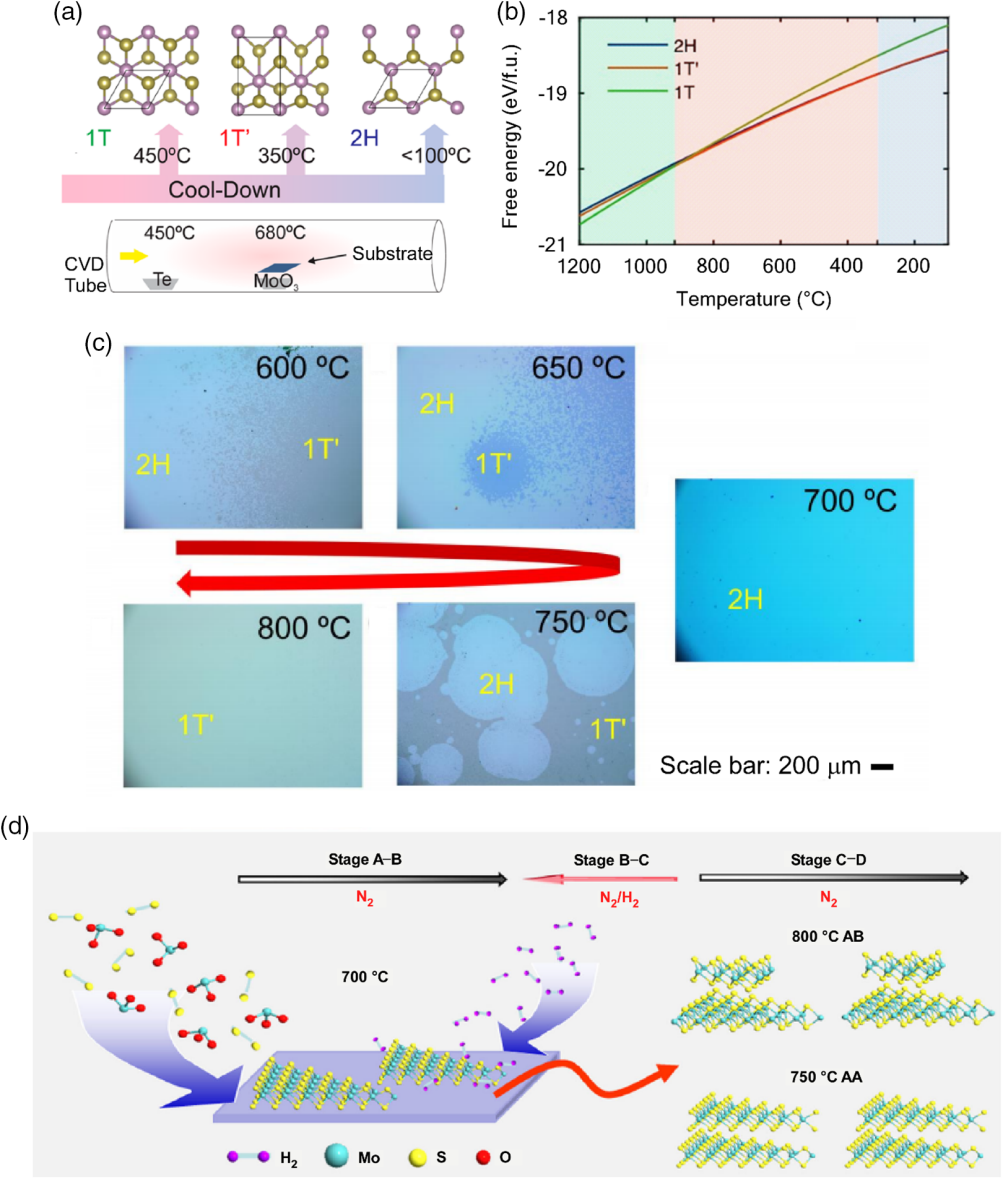
a) Schematic illustration of the CVD setup for MoTe_2_ and the phases obtained by different quenching temperatures. b) Free energy of 1T, 1T′, and 2H phases of MoTe_2_ against temperature plot. a,b) Reproduced with permission.^[^ ^48^ ^]^ Copyright 2016, American Chemical Society. c) Optical images of MoTe_2_ films deposited at different temperatures deposited at between 600 and 800 °C. Reproduced with permission.^[^ ^50^ ^]^ Copyright 2019, American Chemical Society. d) Schematic illustration of the reverse flow CVD process for the deposition of bilayer MoS_2_. Reproduced under the terms of the CC‐BY 4.0 license.^[^ ^53^ ^]^ Copyright 2019, The Authors, published by Springer Nature.

Temperature control is a direct approach to 2D TMD phase engineering, as it is a basic parameter governing the thermodynamics and kinetics of phase transformation. In general, such temperature control for phase‐selective CVD takes advantage of changes in the thermodynamic stability of phases and/or blocks/assists certain kinetic pathways for phase transformation. Temperature control has been primarily performed on Te‐based TMDs because the energy differences among their phases are relatively small compared with other TMDs and are prone to reversal under complex CVD conditions associated with temperature changes. A typical example of temperature‐controlled phase engineering is the work by Empante et al., in which the 2H, 1T, and 1T′ phases of MoTe_2_ were selectively obtained by controlling the growth and quenching temperatures during a one‐step CVD process (**Figure** **8**a).^[^ ^48^ ^]^ MoTe_2_ films were initially grown at 650 or 680 °C targeting the 2H or 1T/1T′ phase, respectively. Then, specialized cooling processes were used to reach the 2H, 1T, and 1T′ phases. Slow cooling to 450 or 350 °C followed by quenching (opening the furnace) produced 1T or 1T′ phases, respectively, while slow cooling to 100 °C before opening the furnace resulted in the 2H phase. These results agree with computed calculations that predict the transition from 2H to 1T′ phase after 310 °C, suggesting that the quenching process should start at 350 °C to completely avoid the 2H phase. Computational studies were also reported; Figure 8b shows the free energy versus temperature plots for all phases; the lowest phase at any temperature is the thermodynamically preferred. These predictions agree with the experimental results in which at low temperature, the 2H phase is preferred and at high temperature 1T phase is dominant.

Sung et al. performed the temperature‐controlled synthesis of 2H and 1T′ MoTe_2_ in a different way by using a salt‐assisted CVD process adding NaCl.^[^ ^21^ ^]^ At the growth temperature of 670 °C, hexagonal or triangular grains corresponding to 2H phase were obtained, while setting the growth temperature to 710 °C led to 1T′ phase rectangular and trapezoidal crystals. At the interval of 670–710 °C, a mixture of 2H and 1T′ phases was deposited and the 2H phase fraction increased along with the temperature. This trend of temperature‐dependent phase stability for CVD‐grown samples is consistent with that reported for MoTe_2_ crystals using the flux growth method, as shown by the phase diagram in Figure 1a in Keum et al.^[^ ^30^ ^]^ In this study, the 2H phase initiates a transition to the 1T′ phase at temperatures higher than 500 °C with a slow cooling process resulting in stable 2H phase. Similar temperature control has also been performed on the CVD of TMD alloys, such as Mo_1–*x*_W_*x*_Te_2_.^[^ ^49^ ^]^ Weyl semimetallic T_d_ phase Mo_0.29_W_0.72_Te_1.99_ nanocrystals were obtained at 820 °C, and Mo_0.32_W_0.67_Te_2.01_ with a mixture of T_d_ and 2H phases was deposited at 760 °C. Herein, the difference in Te content also affected the deposition of different phases because an excess of Te content expands the temperature range for 2H phase formation.

In the case of two‐step CVD, a temperature dependence of phase formation different from the aforementioned one‐step case has been observed.^[^ ^50^ ^]^ MoTe_2_ samples were prepared under the same reaction conditions but at different growth temperatures, by tellurizing predeposited 3 nm Mo films. The mixture of 2H and 1T′ was obtained at 600 °C, and the content of 2H increased with increasing temperature and reached pure 2H at 700 °C. Further higher growth temperature led to the reappearance of 1T′ phase, and the pure 1T′ phase was achieved at 800 °C (Figure 8c). It should be noted that multiple factors, such as temperature, composition, and strain, have contributed to the phase transformation at the same time, while the temperature is the handle to steer the other factors. Details on controlling strain and composition for phase engineering will be discussed separately in following sections. In addition to phase engineering among the intrinsic structures of a layer, temperature control has been shown to influence the CVD of phases with different van der Waals stacking sequences. Chen et al. reported the deposition of 3R phase MoS_2_ and WS_2_ with a small portion of 2H phase at a growth temperature of ≈1050 °C.^[^ ^51^ ^]^ The larger patterns of the deposited film exhibited the 3R phase with a low portion of 2H phase crystals. This distinct behavior of metastable 3R phase formation is considered to be associated with the high temperature used in the reaction process.^[^ ^52^ ^]^ Furthermore, another recent study using a reverse‐flow CVD method also produced MoS_2_ bilayer single crystals with different stacking structures.^[^ ^53^ ^]^ This CVD method involves two deposition stages and reverse‐hydrogen‐flow process, as shown in Figure 8d. The first stage, denoted as A–B, is the occurrence of the first MoS_2_ layer deposition, and the growth of the second layer is achieved in the second stage, C–D. The growth of the first layer was conducted at 700 °C, while a higher growth temperature of 750–800 °C was used in the second stage. The reverse‐hydrogen‐flow process was implemented during the temperature ramp from stage A–B to stage C–D. This reduces the formation of new nucleation centers during the temperature ramp by minimizing the precursor vapor supply so that the second layer grows on the active nucleation centers on top of the first layer.^[^ ^54^ ^]^ Interestingly, the final stacking structure of the bilayers was determined by the growth temperature of the second stage. AA stacking, corresponding to 3R MoS_2_, was obtained at 750 °C, and AB stacking, corresponding to 2H MoS_2_, was obtained at 800 °C.

In contrast to the temperature control at relatively high temperatures noted earlier, very low‐temperature deposition of the metastable 1T phase has been reported to be achieved by a plasma‐enhanced chemical vapor deposition (PE‐CVD) method. Using 1 nm‐thick W film deposited on Si/SiO_2_ and H_2_S as precursors with a growth temperature of 150 °C, the metastable 1T phase of WS_2_ was reported.^[^ ^55^ ^]^ However, the detailed mechanism of such a phase engineering process remains unresolved.

### 4.2 Controlling Precursors

In the CVD‐based phase engineering of 2D TMDs, the type of precursors and the way of supplying them into the reaction chamber are essential parameters that affect the formation of different phases. For instance, in the synthesis of MoTe_2_ films, the tellurization of elemental Mo produces a drastic unit cell volume change from 15.6 Å^3^ (Mo) to 224.62 Å^3^ (MoTe_2_), which results in a large built‐in compressive strain that stabilizes the 1T′ phase, while the conversion from MoO_3_ with a unit cell volume of 202.99 Å^3^ to MoTe_2_ with 224.62 Å^3^ produces insufficient strains to trigger the phase transformation from 2H to 1T′.^[^ 32, 56 ^]^ Recently, Fraser et al. also investigated the effect of transition metal precursors, Mo and MoO_2_, on tuning the phases of MoTe_2_.^[^ ^44^ ^]^ Instead of the commonly used Te powder, FeTe_2_ was used as the source of tellurium. The results revealed that Mo produces 2H phase, which has been hypothetically explained by the preference of the formation of the highly symmetric 2H phase from the highly symmetric crystalline Mo precursor. On the contrary, MoO_2_ produced 1T′ phase, probably because both present the lower monoclinic symmetry, based on a similar argument.^[^ ^44^ ^]^ Stoichiometry/nonstoichiometry of the precursors is also an important factor leading to phase engineering. Tellurization of MoO_3_ at all temperatures leads to 2H phase MoTe_2_ deposition, while MoO_2.0–2.5_ leads to temperature‐ and time‐dependent selection of 2H and 1T′ phases.^[^ ^57^ ^]^ It is noteworthy that the difference in the behavior of MoO_2.0–2.5_ and MoO_3_ lies on the Te vacancy concentration they result, which will be further elaborated in Section 4.3.

**Figure 9:**
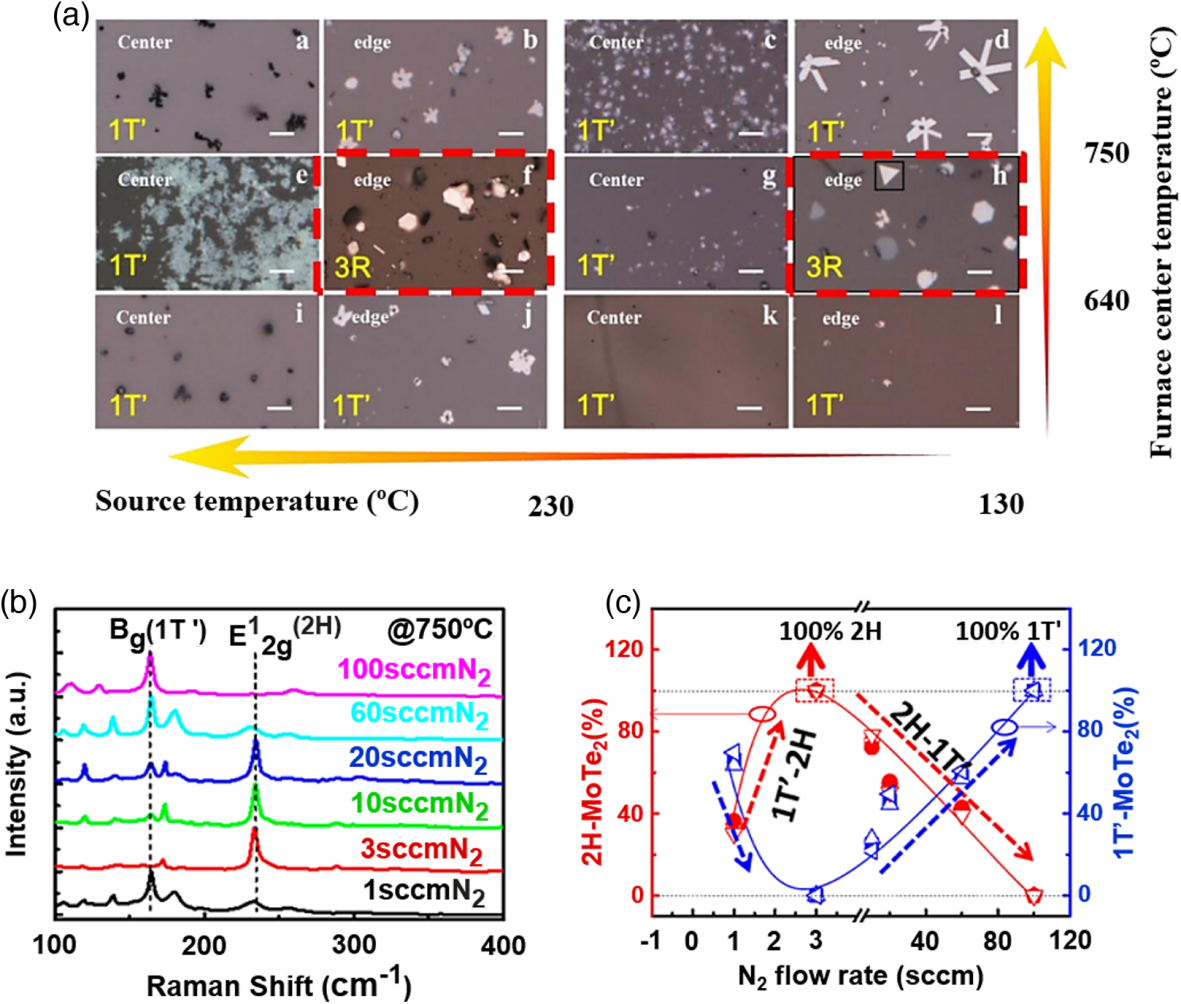
a) Effect of the deposition parameters on the synthesis of 3R‐MoS_2_. The results were obtained at different furnace center temperatures and MoCl_5_ temperatures at the furnace center and edge. Reproduced with permission.^[^ ^58^ ^]^ Copyright 2018, Wiley‐VCH. b) Graph of the phase evolution of MoTe_2_ as a function of the carrier gas flow rate. c) Raman analysis of different MoTe_2_ films grown at different N_2_ flow rates. b,c) Reproduced with permission.^[^ ^22^ ^]^ Copyright 2017, American Chemical Society.

In addition to the phase engineering between 2H and 1T′ for 2D MoTe_2_, the formation of 3R MoTe_2_ by CVD has also been achieved by choosing appropriate Mo precursors.^[^ ^58^ ^]^ The nature of Mo source is critical for the synthesis of MoTe_2_: precursors Mo and MoO_3_ have relatively higher melting points, while the melting point of MoCl_5_ is much lower; the binding energy of Mo–Cl is lower than that of Mo–O. Therefore, MoCl_5_ facilitates the formation of Mo–Te at low deposition temperatures, thereby increasing the possibility of obtaining various metastable phases. The optical images of the films obtained at different deposition temperatures and Mo sublimation temperatures and their corresponding phases are shown in **Figure** **9**a, from which the narrow window for the synthesis of 3R MoTe_2_ can be seen.

Apart from the transition metal precursors, the choice of chalcogen precursors can result in the formation of different phases of TMDs as well. The phase of CVD synthesized NbS_2_ thin films was shown to be directly affected by the chalcogen precursor in the study by Carmalt et al.^[^ ^59^ ^]^ In a two‐step CVD process, the same Nb source (NbCl_5_) reacts with different sulfur precursors, specifically S(SiMe_3_)_2_, *t‐*Bu_2_S_2_, *t‐*BuSH, and HSCH_2_CH_2_SH. The films deposited with HSCH_2_CH_2_SH or *t‐*BuSH lead to the 3R phase in the temperature range of 350–600 °C, while S(SiMe_3_)_2_ or *t‐*Bu_2_S_2_ gives rise to the formation of 1T phase at temperatures between 500 and 600 °C. Such phase‐selective growth of NbS_2_ thin film was considered to be resulted from the fast growth rate when S(SiMe_3_)_2_ and *t*Bu_2_S_2_ (3.2 μm min^−1^ at 600 °C) were used. In contrast, the deposition using HSCH_2_CH_2_SH or *t‐*BuSH at the same temperature showed a much lower growth rate of 0.1 μm min^−1^ which led to the 3R phase. Despite that the phase‐selective deposition has been related to the difference in growth rate when using different chalcogen precursors, the underlying mechanism remains elusive. Further experimental and theoretical analyses elucidating this issue may lead to a more systematic route for phase engineering by the choice of chalcogen precursors.

It should be noted that not only the type of precursor but also the way of supplying the precursors affects the final phase of the material. For example, the chalcogen flow rate plays an important role in the phase engineering of 2D TMDs. In the work by Yang et al., elemental Te and predeposited MoO_3_ film on a Si/SiO_2_ substrate were used as precursors for a two‐step CVD of 2D MoTe_2_.^[^ ^22^ ^]^ Four different phase configurations, including a single‐phase 2H MoTe_2_, a single‐phase 1T′ MoTe_2,_ and two dual‐phase MoTe_2_ with different fractions of 2H and 1T′ phases, were obtained at a fixed growth temperature of 750 °C. Similar phase configurations have also been achieved under different Te supply conditions.^[^ 30, 60 ^]^

The Te supply was regulated by the combination of carrier gas flow rate and tellurium temperature. A mixture of H_2_ (fixed flow rate of 4 sccm) and N_2_ served as carrier gas. The N_2_ flow was varied in the order of 1, 3, 10, 20, 60, and 100 sccm, which resulted in an increasing flow of Te vapor introduced into the system. At a low flow rate of 1 sccm of N_2_, a mixture of 2H and 1T′ phases was observed. With a slight increase in the tellurization velocity by adjusting N_2_ flow from 1 to 3 sccm, 2H phase fraction reaches 100%, while further increasing of N_2_ flow from 3 to 10, 20, 60 sccm results in the formation of dual‐phase MoTe_2_ again with decreasing 2H fraction, until the 1T′ fraction reached 100% at 100 sccm of N_2_ (Figure 9b). Raman spectra of the films grown at these different carrier gas flow rates confirmed the flow rate dependence of phase fractions (Figure 9c). This flow rate dependence was explained by the different strain conditions originated from the different tellurization rates in the CVD process, which is related to the fast volume expansion from MoO_3_ to MoTe_2_ under fast tellurization. Also, another important factor that influences the MoTe_2_ phase is the presence of tellurium vacancies, which is also related to the control of chalcogen supply.^[^ ^61^ ^]^ Deficiency of Te in the MoTe_2_ grown with a low Te supply rate leads to the formation of 1T and 1T′ phases, while a sufficiently high Te supply rate favors the 2H phase.

In fact, controlling the chalcogen supply by adjusting the heating temperature also shows a similar effect. Yoo et al. investigated the phase evolution of MoTe_2_ as a function of Te temperature using elemental Te powder and predeposited Mo film as precursors.^[^ ^62^ ^]^ By placing the precursor in different heating zones, the temperature of Te can be controlled in the range from 485 to 635 °C. It was determined that the Te flux at 635 °C was greatly increased in comparison with that at 485 °C. Raman analyses of the samples grown at different Te heating temperature reveal that 2H MoTe_2_ was obtained at 635 °C, a 2H‐1T′ mixture was deposited at 585 °C, and the pure 1T′ phase with a high concentration of Te vacancies was obtained at 535 and 485 °C. Such Te flux dependence of the multiple phase formation is again related to the aforementioned Te deficiency condition.^[^ ^63^ ^]^

### 4.3 Composition (Alloying and Doping)

Charge doping from the internal structure of the material caused by vacancies is also very essential in influencing the phase stability and phase transformations of 2D TMDs. For instance, if the hexagonal phase of TMDs is stabilized by a certain electron count, then it is expected that vacancy doping can induce not only structural but also electronic instability to the material. Therefore, the controlled introduction of vacancies in CVD processes is an effective strategy for achieving phase‐engineered growth of 2D TMDs. Contrary to removing atoms from the structure, low‐concentration doping of group 6 TMDs (MX_2_, M: Mo, W, and X: S, Se) with group 5–7 elements can also cause phase transformations.^[^ ^64^ ^]^ Such atomic doping occurs as a direct substitution of the atoms in the lattice, or interstitial sites, which may result in an alteration of the initial phase of the material.^[^ ^65^ ^]^ By doping WSe_2_ with transition metals such as V, Nb, Ta, or Cr, the stable 2H phase transforms into the 1T phase.^[^ ^66^ ^]^ It was also reported that the doping of semiconducting WS_2_ with Ta and Nb produces 1T phase (>50%).^[^ ^67^ ^]^

It has been reported that chalcogen vacancies have a great effect on the phase stability. Zhang et al. prepared MoTe_2_ films by using either MoO_2.0–2.5_ or MoO_3_ as the source of Mo in a CVD reaction with tellurium powder.^[^ ^57^ ^]^ The films prepared with MoO_2.0–2.5_ or MoO_3_ presented high and low defects concentration, respectively, which resulted in 2H and 1T′ phases by tellurization at 650 °C. The phase‐selected growth is supported by a previous theoretical study, which shows that a Te monovacancies concentration higher than 3% makes 1T′ phase more stable than 2H phase.^[^ ^63^ ^]^

**Figure 10:**
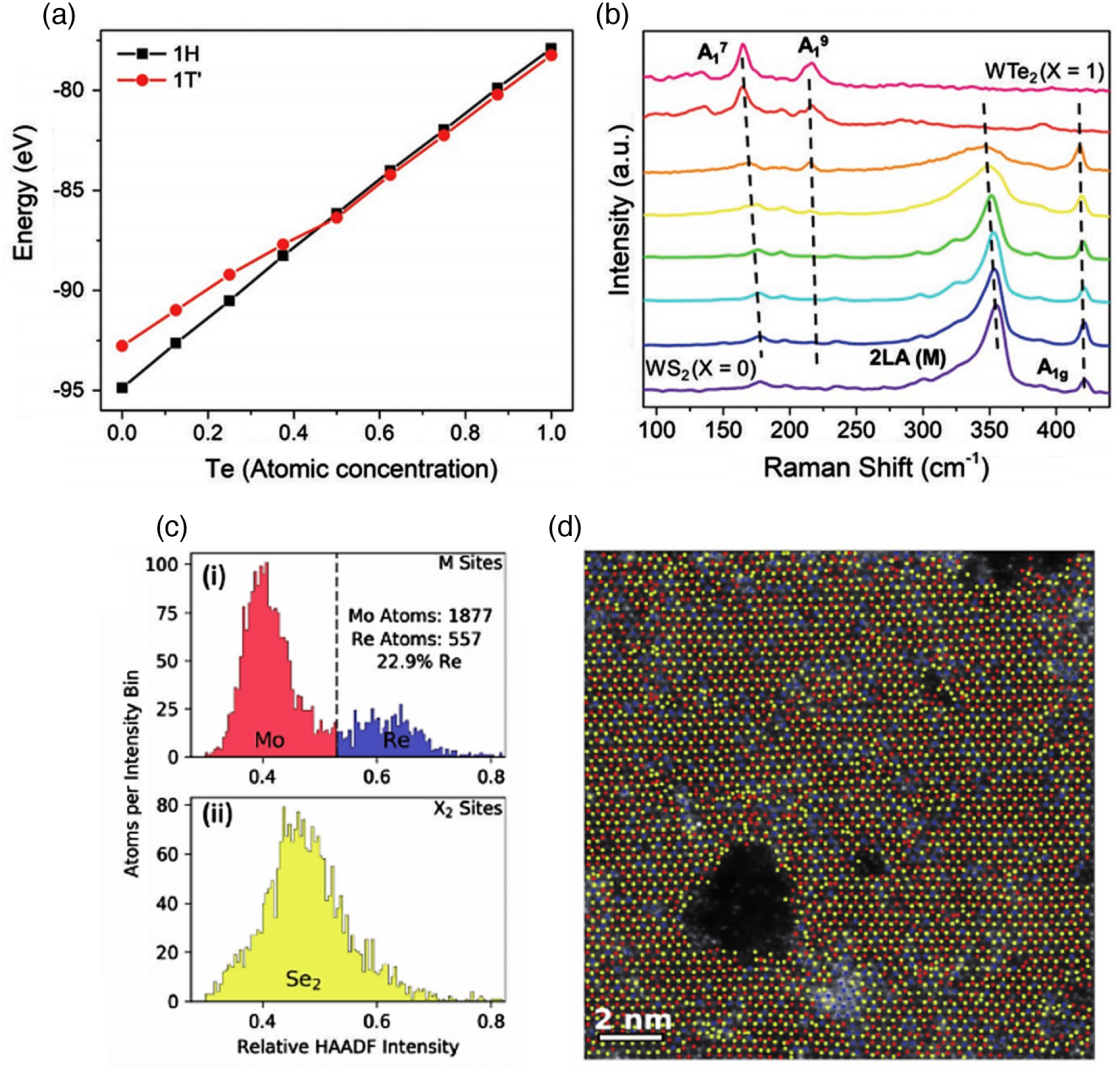
a) Energy shift of WTe_2*x*_S_2(1–*x*)_ alloy versus concentration of Te (*x*). b) Raman spectra of WTe_2*x*_S_2(1–*x*)_ alloys with *x* = 0, 0.04, 0.08, 0.12, 0.16, 0.2, and 0.85, respectively. a,b) Reproduced with permission.^[^ ^68^ ^]^ Copyright 2019, Wiley‐VCH. c) HAADF intensity histograms of metal and chalcogenide sites showing that Re% is 22.9%, confirming a 2H phase. d) Atom labeled image presenting Re doping with uniform distribution. c,d) Reproduced with the permission.^[^ ^69^ ^]^ Copyright 2017, Wiley‐VCH.

It is also possible to modulate the phase by controlling the chemical composition through alloying two TMDs with different structures that allow mutual substitution for either transition metal or chalcogen in a large composition range. Monolayer WTe_2*x*_S_2(1–*x*)_ (*x* = 0–1) alloys with controlled phases have been reported.^[^ ^68^ ^]^ Because WTe_2_ and WS_2_ have ambient stable with 1T′ and 2H structures, respectively, the phase of the monolayer WTe_2*x*_S_2(1–*x*)_ alloys can be tuned from 1T′ to 2H by varying the chalcogen composition. The semiconducting 2H WTe_2*x*_S_2(1–*x*)_ (*x* = 0.08) was formed at a Te:S = 1:1 ratio and the 1T′ metallic WTe_2*x*_S_2(1–*x*)_ (*x* = 0.85) required a Te:S = 10:1 ratio. By comparing the energies of 2H and 1T′ phases for different Te fractions, first‐principle calculations further predicted a critical Te fraction (*x*_c_) for inducing the phase transformation to be *x*_c_ = 0.45 (**Figure** **10**a,b). A similar strategy can be applied to the case of alloying by mixing different transition metal elements. For example, the CVD‐grown Mo_1–*x*_Re_*x*_Se_2_ alloy shows a transition between the 2H (MoSe_2_) and 1T′ (ReSe_2_) phases at intermediate compositions.^[^ ^69^ ^]^ Both experimental results and DFT calculations show that the 2H phase is favored below 40% Re, while the 1T′ phase is favored at higher Re concentrations. The phase engineering mechanism can be simply understood from the addition of extra electrons from the Re dopants into the matrix MoSe_2_. This electron fills the higher energy level in the trigonal prismatic coordination of the 2H phase destabilizing the 2H phase of MoSe_2_. On the contrary, the octahedral coordination in the 1T′ phase gets stabilized by filling its lower energy state with the extra electron. HAADF intensities histograms of Re, Mo, and S obtained from a Re‐doped MoSe_2_ sample quantified the Re concentration as 22.9% which confirms the 2H nature of that specific area of the **s**ample (Figure 10c,d). When the Re atoms outnumber the Mo atoms making the doping concentration of Re higher than 40%, 1T′ phase becomes the preferred structure.

### 4.4 Controlling Reducing Atmosphere

In the CVD growth of 2D TMDs, H_2_ has been commonly used as a reducing agent (or activator/etchant) to assist the reaction between the transition metal and chalcogenide precursor. It has been reported that it also plays an active role in the phase control of deposited 2D TMDs.^[^ ^70^ ^]^ For the specific case of the CVD growth of transition metal ditellurides, the reaction between H_2_ and Te powder results in the formation of an intermediate product H_2_Te, which reacts with the transition metal precursor to produce the target TMD.^[^ ^71^ ^]^ Zhou et al. reported the CVD deposition of T_d_ WTe_2_ through the two‐step reaction of W films with Te.^[^ ^72^ ^]^

(1) $$H_{2}(\text{gas})+\text{Te }(\text{vapour})↔H_{2}\text{Te }(\text{gas})$$

(2) $$H_{2}\text{Te}+\text{W }\to {\text{WTe}}_{2}+{\text{ H}}_{2}$$

**Figure 11:**
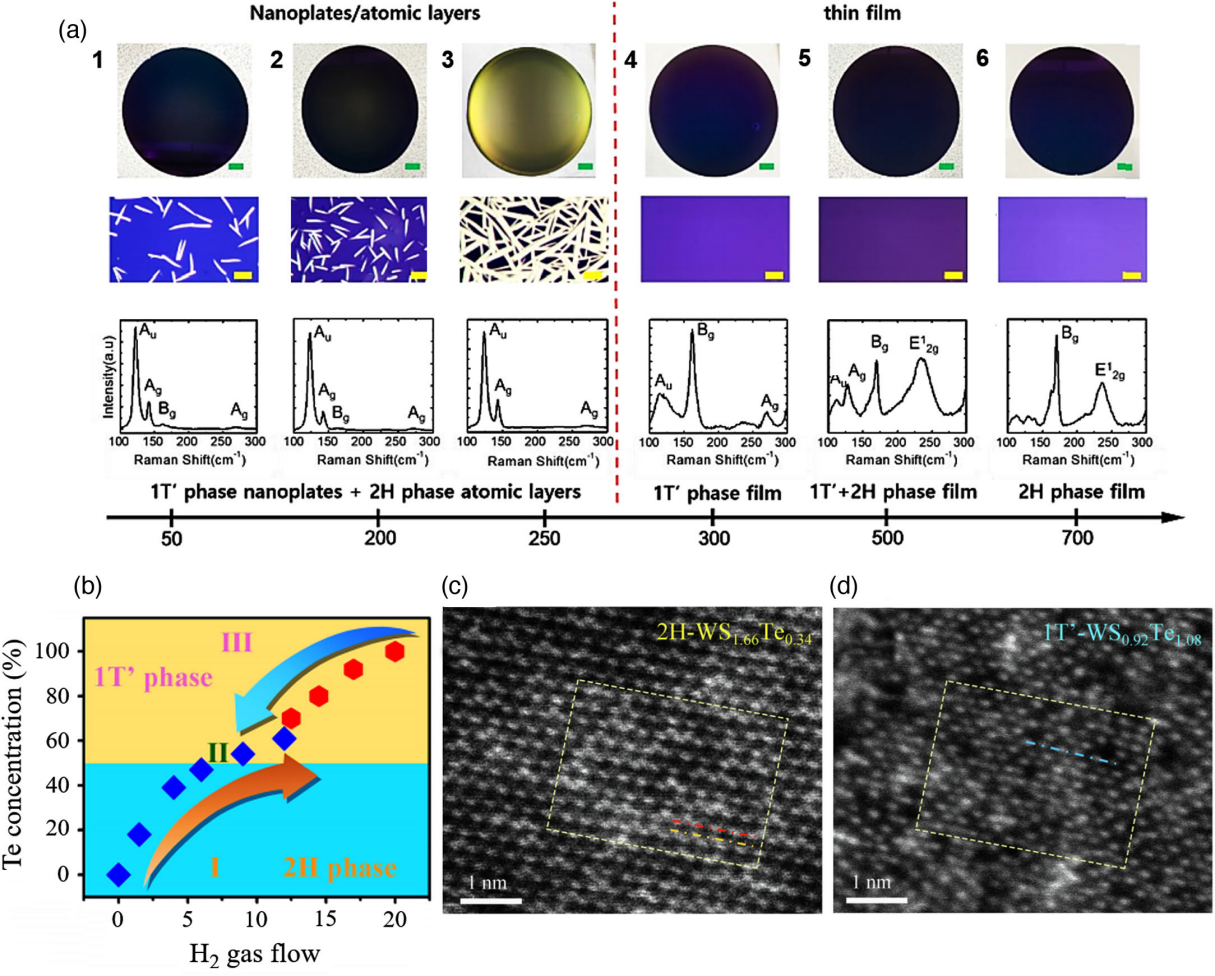
a) Optical images and Raman spectra obtained from samples prepared with 1) 50 sccm, 2) 200 sccm, 3) 250 sccm, 4) 300 sccm, 5) 500 sccm, and 6) 700 sccm of H_2_. The green and yellow scale bars represent 2 and 5 μm, respectively. Reproduced with permission.^[^ ^73^ ^]^ Copyright 2019, American Chemical Society. b) Phase diagram of WS_2(1−*x*)_Te_2*x*_ alloys with Te% as function of the H_2_ gas flow. c–d) HAADF‐STEM images of alloys with 2H and 1T′ phases, respectively. Insets show the FFT patterns from the 2H and 1T′ phases. b–d) Reproduced with permission.^[^ ^74^ ^]^ Copyright 2019, Elsevier B.V.

By adjusting the H_2_ molar flow rate, phase‐selective synthesis of MoTe_2_ thin film can be realized.^[^ ^23^ ^]^ Atomically, thin 1T′ and 1T′/2H dual‐phase MoTe_2_ were grown at a low H_2_ flow rate (indicating a low H_2_/Te ratio of the atmosphere), while 2H phase MoTe_2_ was obtained at a high H_2_ flow rate. The role of H_2_ in the CVD reaction has been previously explained by DFT calculations: the adsorption of H_2_ molecules on the surface of MoTe_2_ promotes the semiconducting 2H phase, while atomic H adsorption favors the 1T′ phase.^[^ ^33^ ^]^ Moreover, Lee et al. have also reported a similar synthesis confirming the mechanism.^[^ ^73^ ^]^ As shown in **Figure** **11**a, only a mixture of 1T′ nanoplates and 2H atomic layers was obtained at the H_2_ flow rate between 50 and 250 sccm, while pure 1T′ and 2H films were grown at a flow rate of 300 and 700 sccm, respectively.

The CVD reaction between K_2_MoS_4_ and H_2_ produces K_*x*_MoS_2_ results in 1T′ and 2H phases. The reaction was controlled via changing the composition of the reducing atmosphere, specifically by mixing H_2_ into Ar carrier gas with various H_2_ fractions ranging from 0 to 10 sccm. Particularly, a high concentration of H_2_ in the carrier gas gives rise to 1T′ phase formation, while an inert atmosphere with pure Ar results in the 2H phase. Alternatively, the intermediate concentrations of H_2_ results in the mixture of 2H and 1T′ phases, and the unique 1T′/2H bilayer structure can be formed at higher temperatures as evidenced by STEM atomic structure observations. Such a phase diagram as a function of reducing atmospheric conditions (H_2_ concentration in the carrier gas flow) and reaction temperatures may act as a map to pursue specific phases by this CVD method. The same study also reported the deposition of 1T′ WS_2_ films by a very similar CVD procedure involving the reaction of the precursor K_2_WS_4_ under a reducing atmosphere (12% of H_2_) at 850 °C.

The phase of TMDs alloys can also be tuned by controlling the flow of H_2_ gas into the CVD reactor. WS_2(1−*x*)_Te_2*x*_ alloys were prepared by H_2_ flow‐controlled CVD method with the assistance of NaCl as catalyst to facilitate the reaction.^[^ ^74^ ^]^ By altering the H_2_ flow into the reactor, the composition of the alloys was modulated. With a H_2_ gas flow of 0–6 sccm the final phase of the alloys was determined as 2H with *x* ≤ 0.5, and when the flow was increased up to 9–12 sccm 1T′ phase was obtained with *x* ≥ 0.5. Figure 11b shows the phase diagram of WS_2(1−*x*)_Te_2*x*_ alloys with Te% as a function of the H_2_ flow. As Te presents a high melting point, the amount of H_2_ in the reaction is crucial to control the Te composition. Figure 11c,d shows the HAADF‐STEM images of the 2H (WS_1.66_Te_0.34_) and 1T′ (WS_0.92_Te_1.08_) alloys, respectively. The different atomic structure of the two phases can be easily identified. The insets in each image show the FFT patterns in which the 2H phase clearly show its characteristic hexagonal structure while that of the 1T′ phase presents rectangular structure.

### 4.5 Introducing Catalysts

Incorporating catalytic effects into the CVD process is another effective approach to achieve phase‐engineered growth of 2D TMDs. Catalyst or promoters, such as alkali metal halides (NaCl, KCl, KI), NaOH, and sodium cholate (C_24_H_39_NaO_5_), have been widely used to facilitate or change the manner of CVD growth of 2D TMDs.^[^ 14, 75 ^]^ In particular, NaCl can universally promote the reaction rate by decreasing the melting point of the precursors and facilitating the formation of intermediate products in the growth of a variety of 2D TMDs and their alloys.^[^ ^14^ ^]^ Significantly, the involvement of these promotors in the CVD process also has notable effects in stabilizing metastable phases.

**Figure 12:**
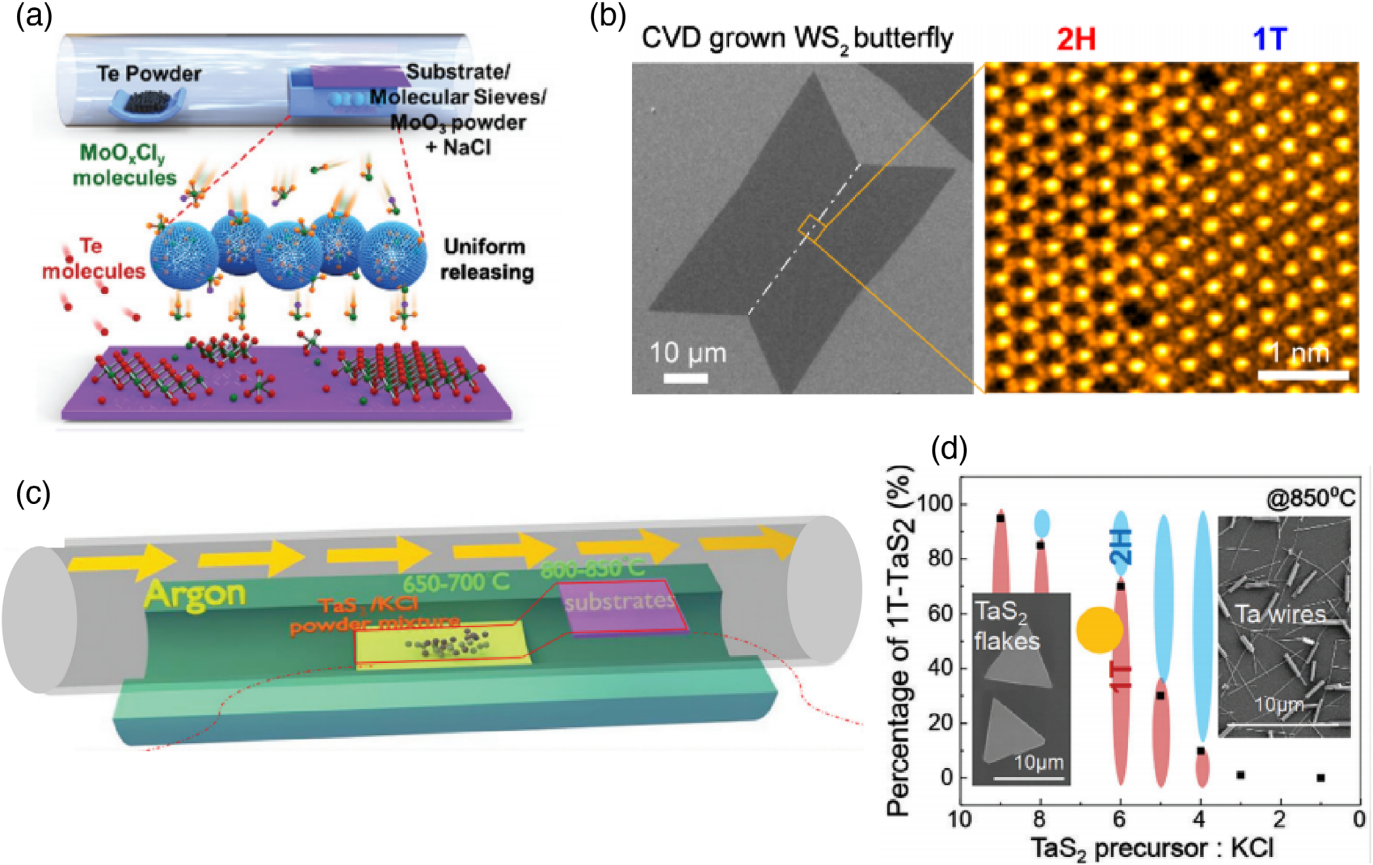
a) Schematic representation of CVD grown 1T′ MoTe_2_ assisted by molecular sieves. Reproduced with permission.^[^ ^77^ ^]^ Copyright 2020, The Royal Society of Chemistry. b) SEM image of a butterfly‐like WS_2_ flake with an ADF image taken in the grain boundary of different phases. Reproduced with permission.^[^ ^78^ ^]^ Copyright 2018, American Chemical Society. c) Schematic illustration of the CVD process for the synthesis of 1T TaS_2_. d) TaS_2_ precursor:KCl ratio versus percentage of 1T TaS_2_ diagram. c,d) Reproduced with permission.^[^ ^79^ ^]^ Copyright 2019, The Royal Society of Chemistry.

Zhang et al. have reported that addition of potassium iodide (KI) as a catalyst in a one‐step CVD growth of MoTe_2_ results in the formation of the 1T′ phase which is originally ambient metastable.^[^ ^76^ ^]^ The substrate was placed over the mixture of Mo precursor (MoO_3_) and the catalyst, using a mixture of Ar/H_2_ as carrier gas. DFT calculations demonstrated that when iodine atoms are adsorbed to (or involved in) the MoTe_2_ lattice, the Mo—Te bond length changes, making 1T′ phase more stable than 2H phase. Moreover, the adsorbed iodine atoms escape from the deposited 1T′ MoTe_2_ at high temperatures, leaving uniform 1T′ phase flakes with minimized iodine residues. Alternatively, CVD synthesis of 1T′ MoTe_2_ had been reported by using salts such as NaCl as well as molecular sieves.^[^ ^77^ ^]^ The role of NaCl as an assisting agent is to increase the vapor pressure of the Mo precursor at lower temperatures by forming low‐melting‐point MoO_*x*_Cl_*y*_. At low temperatures, the molecular sieves control the release of the Mo precursor by acting as a physical barrier. When the temperature is increased, the vapors of the Mo precursor can diffuse into the pores of the molecular sieve. This procedure greatly benefits the formation of uniform TMDs growth, as shown in **Figure** **12**a.

Fe_3_O_4_ and NaCl, have also served as promoters and catalysts in the one‐step CVD growth of 1T WS_2_, playing a significant role in the formation of the metallic phase.^[^ ^78^ ^]^ The Fe_3_O_4_ was mixed with the WO_3_ precursor, while the NaCl was placed separately at the upstream side of WO_3_. A special butterfly‐shaped double‐grain monolayer WS_2_ or the triangle‐shaped single‐grain monolayer WS_2_ flakes were obtained. Interestingly, almost all the butterfly‐shaped flakes contain the 2H phase in one wing‐shaped grain and 1T phase in the other, as evidenced by the SEM and ADF‐STEM images in Figure 12b. To understand the formation of the 1T phase, the CVD process was stopped at the nucleation stage; it was determined by XPS analysis that a volatile intermediate Na_*x*_WO_*y*_ is produced. The formation of nuclei of two different phases can be attributed to a nonhomogenous charge doping produced by the Na atoms. Furthermore, DFT calculation shows that the presence of alkali metal atoms on the surface of the material can lead to a decrease in the formation energy of the 1T phase by 0.24 eV. Fe_3_O_4_ induces the growth of butterfly‐shaped 1T/2H flakes with Fe atoms combined into the WS_2_ lattice. Nevertheless, its catalytic properties remain unclear.

In addition to halogen, the alkaline metal elements in the promoters also affect the phase selection in the CVD growth, which is very similar to the alkaline‐intercalation‐induced 1T phase formation in the chemical exfoliation process. In the potassium halide (KI and KCl)‐assisted synthesis of TMDs, phase‐engineered growth has been achieved due to the transfer of excessive electrons from potassium to the 2D TMDs which stabilizes the metallic 1T phase. Figure 12c schematically shows the one‐step CVD growth of metastable 1T phase TaS_2_.^[^ ^79^ ^]^ TaS_2_ and KCl powders were mixed and loaded into an alumina boat for the CVD reaction. With the TaS_2_:KCl ratio changing, the concentration of K^+^ intercalated into the deposited TaS_2_ changes, thereby controlling the fraction of 1T and 2H phases. Accompanied with this phase fraction change, the morphology of the deposited flakes also shows a distinct trend of evolution, as shown in Figure 12d. When the ratio is between 1 and 3, only Ta wires can be obtained. Increasing the ratio to 6–7 leads to the deposition of TaS_2_ flakes with an increased fraction of 1T phase but with a messy morphology. Finally, further increasing the ratio up to 10 results in TaS_2_ flakes containing mostly 1T phase with well‐defined triangle shape.

### 4.6 Controlling Strain

The phase engineering based on strain control is of great importance because it couples mechanical changes with abrupt property changes, e.g., a semiconductor–metal transition for group 6 TMDs. Both tensile and compressive strains have been theoretically and experimentally demonstrated to give rise to phase transformations of 2D TMDs. An early computational work by Duerloo et al. predicted the 2H to 1T′ phase transformation under in‐plane uniaxial and biaxial tensile strains for Mo‐ and W‐based monolayer TMD except for WTe_2_ which has an intrinsically stable 1T′ phase.^[^ ^32^ ^]^ In‐plane compressive strain along the armchair direction can cause a 1T′ to 2H phase transformation in monolayer WTe_2_.^[^ ^32^ ^]^ Experimentally, various methods of applying strain have been developed, mostly relying on the transfer of 2D TMDs to flexible substrates or patterned substrates with protrusions/cavities.^[^ ^80^ ^]^ Although these procedures were successful in applying external strains to TMDs macroscopically, inhomogeneity issues arising from the transfer process, such as air bubbles and wrinkles, are considered to impair the material performance. To avoid these complications, inducing strains during the in situ growth of TMDs in a CVD process can be an effective approach.

**Figure 13:**
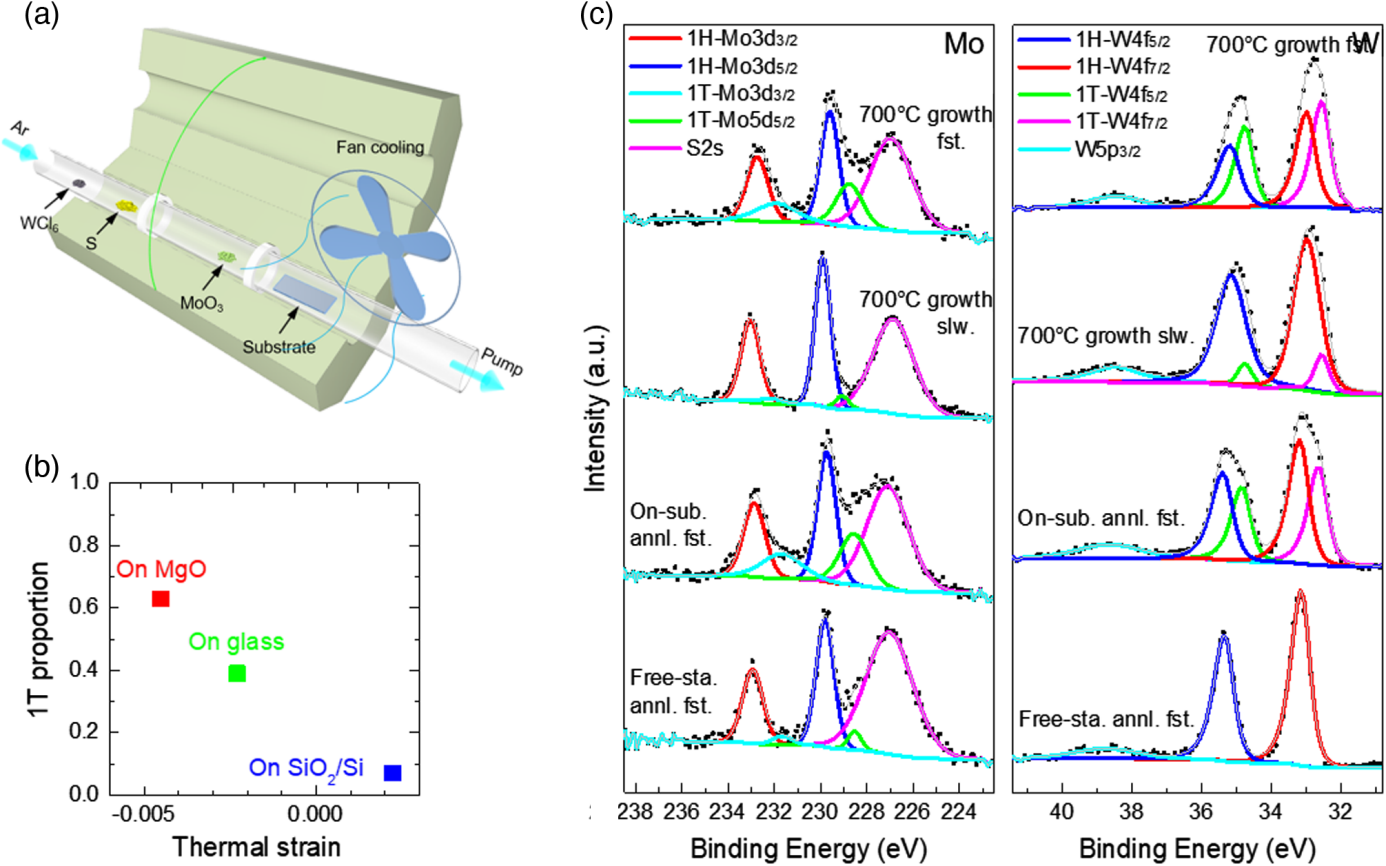
a) Schematic representation of the CVD setup used for the synthesis of Mo_1−*x*_W_*x*_S_2_. An electric fan was used to induce fast cooling in the sample, when the fan was not used the cooling was slow. b) Graph of 1T phase proportion versus thermal strains from different substrates. c) XPS results of the Mo_1−*x*_W_*x*_S_2_ films grown on glass at 700 °C followed by an annealing process at 500 °C while on the substrate, or a free‐standing annealing at 500 °C. The terms “Fst.,” “slw.,” “on‐sub.,” “free‐sta.,” and “annl.” are assigned to “fast cooling,” “slow cooling,” “on‐substrate,” “free‐standing,” and “annealing,” respectively. a–c) Reproduced with permission.^[^ ^81^ ^]^ Copyright 2018, American Chemical Society.

In contrast to the strain control strategy via the chemical reaction process of a CVD, in‐plane strains can be directly applied to the 2D TMDs in the cooling process by taking advantage of the thermal expansion coefficient (TEC) mismatch between the 2D TMD and the substrate. As no strain is present in the monolayer TMD flakes or films at the growth temperature right after deposition, the following cooling process applies thermal strains to the TMD layers as a consequence of the TEC difference. In particular, compressive strain, which is usually less convenient to be generated by other means, can be achieved by using substrates with a large TEC with this method. Wang et al. confirmed that the phase transformation from 2H to 1T can be introduced via such compressive thermal strain in the cooling process for monolayer Mo_1–*x*_W_*x*_S_2_ alloys; the schematic representation of the CVD setup is shown in **Figure** **13**a.^[^ ^81^ ^]^ Monolayer MoS_2_ and WS_2_ have TECs of 7.1 and 6.5 × 10^−6^ K^−1^, respectively, and their alloys were considered to have similar TEC values.^[^ 81, 82 ^]^ Different thermal strain values can be obtained by using SiO_2_/Si (TEC = 3.5 × 10^−6^ K^−1^), soda‐lime glass (TEC = 9.0 × 10^−6^ K^−1^), and MgO (TEC = 1.5 × 10^−5^ K^−1^) substrates.^[^ ^81^ ^]^ Figure 13b shows a graph of 1T phase fraction versus thermal strain in the monolayer Mo_1–*x*_W_*x*_S_2_ (*x* ≈ 0.5) grown on these substrates under the same conditions. A clear trend can be seen that larger compressive thermal strain results in higher 1T phase fraction (MgO and soda‐lime glass) while tensile strain hardly causes 1T phase formation (SiO_2_/Si). These results were obtained from the XPS analysis, as shown in Figure 13c. By using an electric fan, a faster cooling rate was achieved, minimizing the effect of thermal annealing during the cooling process, thereby promoting the generation of higher thermal strains in CVD‐grown samples. Intriguingly, compared with Te‐based group 6 TMDs, these S‐based group 6 TMDs are considered to be more difficult to perform phase engineering because of the larger energy difference between 2H and 1T phases as well as the larger energy barrier for 2H‐to‐1T phase transformation. Therefore, the thermal strain‐induced phase transformation in the CVD cooling process has proven to be an effective strategy for phase engineering.

## 5 Applications of Phase‐Engineered 2D TMDs Prepared by CVD

**Figure 14:**
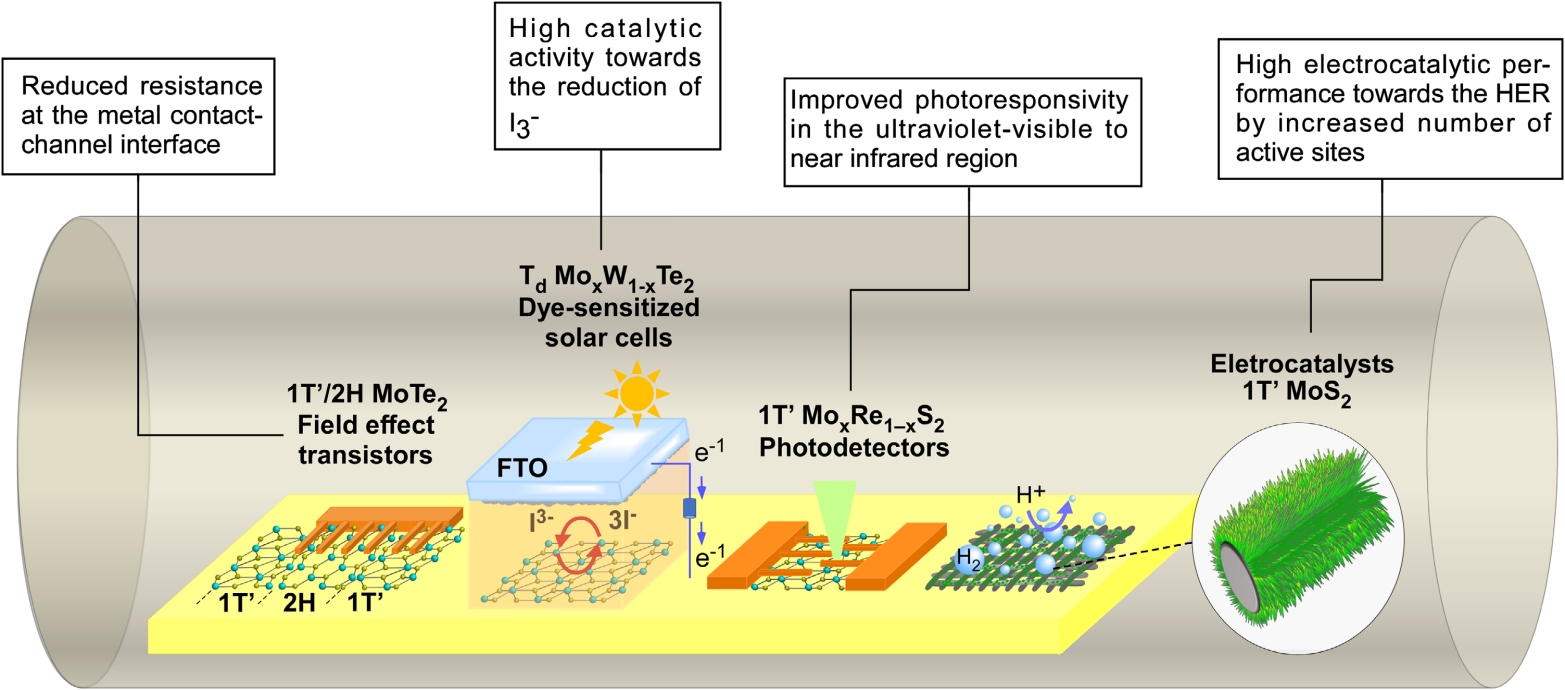
Schematic illustration of electronic, optoelectronic, and electrocatalytic applications for CVD phase‐engineered 2D TMDs.

When taking the crucial step from fundamental research to industrial applications, the production of large‐area 2D materials with phase versatility is desperately desired. In this sense, the CVD method prevails as a potential technique to provide large‐scale or wafer‐scale TMD deposition which can hardly be realized by the conventional mechanical exfoliation as mentioned before. Recent studies have shown the outstanding performance of these CVD‐grown TMDs in various fields, such as electronic/optoelectronic devices and energy harvesting, due to the presence of a particular single phase or the combination of multiple phases. With the outline shown in **Figure** **14**, this section summarizes representative works on the application of CVD‐based phase‐engineered TMDs in FETs, photovoltaic devices, photodetectors, and HER electrocatalysis.

### 5.1 FETs

A FET is a device that uses an electric field to control the flow of electrical current and manipulate the electronic signals. The device consists of three terminals: source, drain, and gate. Electrons or holes are the charge carriers that move from the source to the drain via an active channel, and the flow of charge carriers is controlled by the gate voltage. FETs are the pillar of electronics, and the improvement of their performances is greatly quested by modern technology.

**Figure 15:**
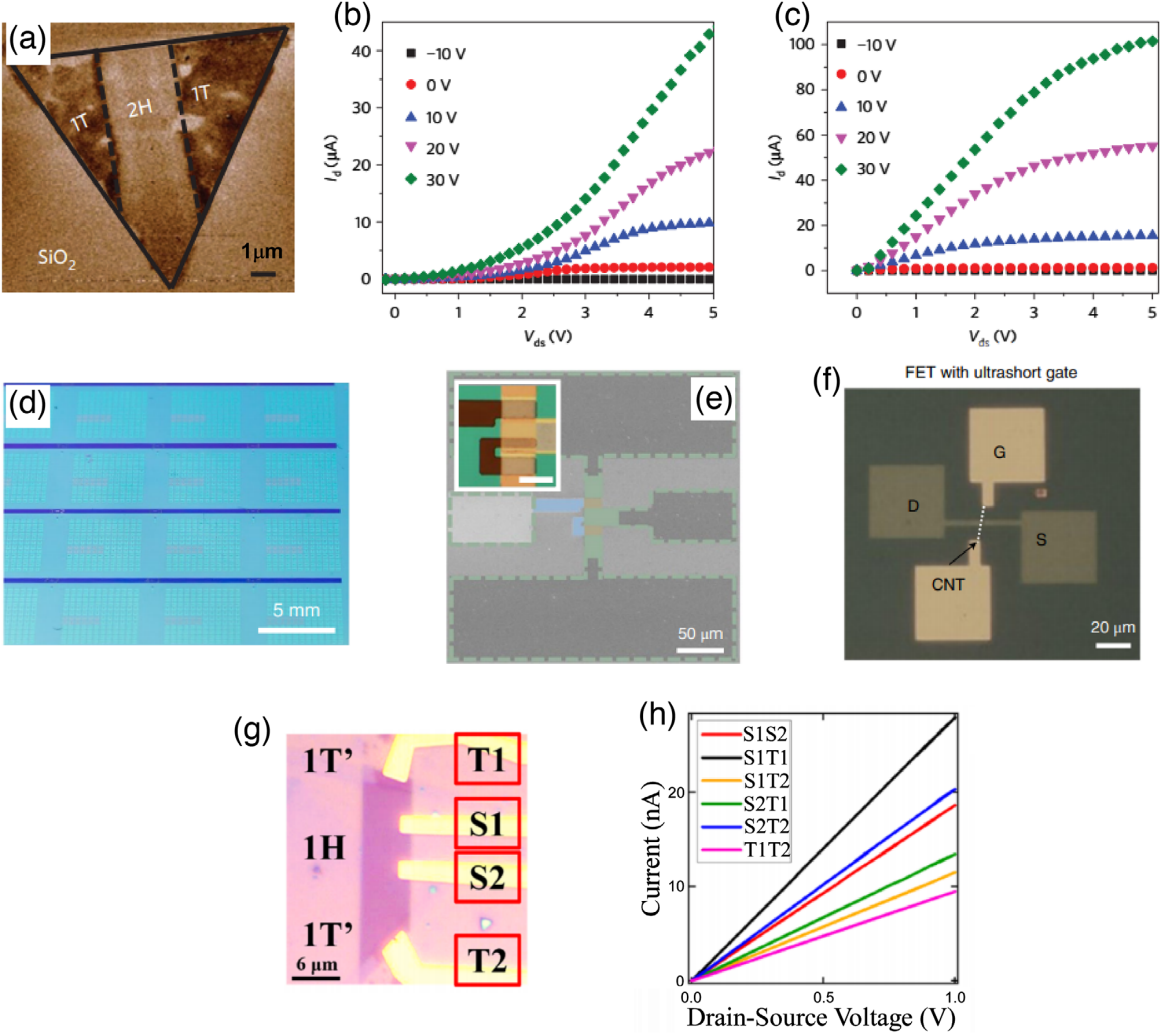
Phase‐engineered 2D TMD‐based FET devices. a) Electrostatic force microscopy image of a MoS_2_ nanosheet shows different phases, 2H phase presents bright color, and 1T phase exhibits dark color. b,c) Output characteristics devices with Au and 1T MoS_2_ electrodes, respectively. *V*_ds_ = 5 V and *V*_gs_ goes from −10 to 30 V. a–c) Reproduced with permission.^[^ ^5^ ^]^ Copyright 2014, Springer Nature. d) Optical image of ≈1500 devices synthesized in a centimeter scale. e) SEM image of the MoTe_2_ device with 2H MoTe_2_, 1T′ MoTe_2_, and WTe_2_ represented by yellow, green, and blue colors, respectively. The inset is an optical image of the device. f) Optical image of a representative MoTe_2_ FET with a CNT gate. d–f) Reproduced with permission.^[^ ^57^ ^]^ Copyright 2019, Springer Nature. g) Optical images of a 1T′/2H/1T′ MoTe_2_ device and its connections. h) Room temperature *I − V* characteristics of different connection pairs. g,h) Reproduced with the permission.^[^ ^87^ ^]^ Copyright 2017, American Chemical Society.

Semiconducting TMDs are promising channel materials for FETs with high on/off current ratios and low off currents.^[^ ^83^ ^]^ Nevertheless, the resistance when carriers pass through the metal contact–semiconductor channel interface, known as the contact resistance, may obstruct the overall carrier flow and limit the performance of the TMD FET devices.^[^ ^84^ ^]^ Much effort has been made to overcome this problem by choosing the metal pad materials with appropriate work functions and/or preferable interface structures.^[^ ^85^ ^]^ It is interesting to note that the presence of semiconducting and metallic regions in the same material even down to the monolayer thickness endows TMDs with a structurally coherent semiconductor–metal lateral homojunction with low contact resistances at the metallic pad/channel interfaces. In particular, a pioneer work by Kappera et al. has demonstrated the significant reduction of contact resistance when an lithium intercalation‐induced 1T phase is placed between the Au pad and the semiconducting 2H MoS_2_ channel (**Figure** **15**a).^[^ ^5^ ^]^ By extrapolating device resistance‐channel length relation, the contact resistances of the two devices with and without intermediate 1T phase show a difference of a factor of five, demonstrating the effective reduction of contact resistance by phase engineering. Concomitantly, a significant improvement in the FET switching behaviors, characterized by larger on–off ratio, higher mobility, and lower subthreshold swing, was achieved (Figure 15b,c).

Benefited from the capability of incorporating phase engineering into CVD growth of TMDs, FETs can be fabricated on phase‐engineered TMDs with as‐grown high‐quality semiconductor–metal interfaces. For this application, MoTe_2_ is the most intensively studied of all TMDs due to the low energy difference between its 2H and 1T′ phases. Ma et al. fabricated FETs on the CVD‐grown dual‐phase MoTe_2_ and studied their transport properties with different junction styles.^[^ ^86^ ^]^ Specifically, three kinds of devices were prepared: 1) 2H channel with Ti/Au metal electrodes attached to the same phase, 2) 1T′ channel with electrodes attached to the same 1T′ phase, and 3) 2H channel with metal electrodes contacted to the abutting 1T′ regions. The contact resistance at the 1T′‐only and 2H‐only devices was measured and the energy barrier at the interface of 2H/1T′ homojunction was also studied. The resistance at the interface of Ti/Au and 1T′ MoTe_2_ (at zero back‐gate voltage) was determined as 0.47 ± 0.03 kΩ μm, while the contact resistance of the interface of Ti/Au and 2H MoTe_2_ was found to be 15.6 ± 0.58 MΩ μm at back‐gate voltage = −100 V. The reduction of the contact resistance when using 1T′ semimetallic interfaces is also clear in the drain current versus drain‐to‐source voltage plot obtained from the evaluation of 2H‐only and 2H/1T′ devices. The performance was measured at −196 and 27 °C, with the 2H/1T′ interface showing a linear output, a clear sign of ohmic‐like behavior. In contrast, the 2H‐only device presented nonlinearity that was even more noticeable at low temperatures, suggesting a Schottky barrier at the metal pad/2H region interface.

Zhang et al. have made a breakthrough progress on the application of CVD‐based phase engineering toward the scalable electronic applications.^[^ ^57^ ^]^ The patterned 1T MoTe_2_ electrodes and 2H MoTe_2_ channels in a single CVD growth avoid material degradation and interfacial pollution and provide covalent connections between channels and electrodes, greatly improving the electrical performance. Transport measurements revealed the p‐type behavior of such 1T′ MoTe_2_‐contacted FET with an on/off current ratio of 10^3^ and a mobility (≈50 cm^2^ V^−1^ s^−1^) 25 times higher than that of the Pd‐contacted FET. The Schottky barrier was also reduced from ≈200 meV (Pd contacts) to ≈20 meV (1T′ MoTe_2_ contacts). In addition, using HfO_2_ as gate dielectrics, further improvements were made on the on/off ratio (reaching 10^5^) and mobility (reaching ≈130 cm^2^ V^−1^ s^−1^), highlighting the high quality and performance of dual‐phase MoTe_2_ FETs prepared by CVD. More importantly, with these metallic 1T′ and semiconducting 2H components, integrated devices including arrays of logic inverters and radiofrequency (RF) transistors were also fabricated in centimeter scale (Figure 15d,e). Moreover, FETs with ultrashort (4 nm) gate lengths by using CNTs as the gate electrodes have been demonstrated to exhibit excellent switching characteristics with a subthreshold swing of ≈73 mV dec^−1^ (Figure 15f). Furthermore, by covering the first‐layer dual‐phase FET devices with HfO_2_ and depositing a second layer of FETs on top, 3D integration in two levels was achieved. The FETs in the two levels were shown to maintain high and similar on/off ratios (≈10^5^) and mobility (≈30 cm^2^ V^−1^ s^−1^), manifesting the robustness of using the CVD‐based phase‐engineered growth for multilevel integrated circuit fabrication.

CVD also shows great potentials in fabricating FET devices based on phase‐engineered heterostructures. Naylor et al. demonstrated the CVD growth of the metallic 1T′ MoTe_2_‐semiconducting MoS_2_ in‐plane heterostructure and a transistor device was fabricated using this dual‐phase material (Figure 15g).^[^ ^87^ ^]^ *I–V* characteristics were measured with electrodes connected to different 2H MoS_2_ and 1T′ MoTe_2_ regions (Figure 15h), showing distinct electrical transport behaviors of different phases/materials and interfaces. Thus, CVD manifests its capability to grow high‐quality phase‐engineered TMD heterostructure films for electronic applications.

It is also important to note that phase engineering of TMDs driven by alloying enables the modulation of the electrical conduction in a FET device. CVD‐grown WS_2(1–*x*)_Te_2*x*_ films were prepared with a Te composition of *x* = 0–1.0.^[^ ^74^ ^]^ Backgate FETs were fabricated with films of different Te and S compositions, i.e., semiconducting WS_2_ and WS_1.6_Te_0.4_ and semimetallic WTe_2_ and WS_0.4_Te_1.6_. The 2H phase devices showed characteristic n‐type performances, while 1T′ alloys did not show drain current dependency. For 1T′ phase devices at a drain voltage of 1 V, the drain current of 1T′ WTe_2_ has considerably increased compared with 1T′ WS_0.4_Te_1.6_. This metallic behavior can be modulated by the content of S in the alloy.^[^ ^88^ ^]^

### 5.2 Photovoltaic Cells

Artificial photosynthesis can be associated with the technology behind dye‐sensitized solar cells (DSSCs) because it resembles the natural process in which plants absorb energy from sunlight. DSSC represents an eco‐friendly process to produce clean energy without using toxic materials. The main components of the device are as follows: 1) porous layer of dye‐sensitized semiconducting TiO_2_ as the photosensitive material, 2) electrolyte, and 3) counter electrode (CE). These materials are usually sandwiched between two glass plates coated with conductive fluorine‐doped tin oxide (FTO) as anode and cathode.^[^ ^89^ ^]^ The whole process is conducted involving all the components. Recently, the high catalytic activity of the CE toward the reduction of I_3_^−^ has become an important research area because the CE executes two important tasks: it catalyzes the reduction reaction of the electrolyte (I_3_^−^ to I^−^) and also collects the electrons from the external circuit carrying them back to the dye.^[^ ^90^ ^]^ CEs are commonly made of a thin film of Pt and although its performance is very efficient, its high cost restricts further development. Therefore, to develop alternative and cheaper materials to substitute Pt electrodes has become a priority.^[^ ^91^ ^]^

CVD‐grown TMDs have proven their efficiency as CE for a DSSCs.^[^ ^92^ ^]^ Recently, CE based on different TMDs polytypes has been recently studied such as 2H‐WS_2_/1T′‐MoTe_2_ heterostructures synthesized by a sputtering‐CVD combined technique.^[^ ^93^ ^]^ For this application, it is crucial to obtain a metallic phase of MoTe_2_, which provides better catalytic performance than its semiconducting polytype as reported in previous electrochemical studies.^[^ ^94^ ^]^ Therefore, when a specific phase is required, phase engineering by CVD becomes a very important tool.

**Figure 16:**
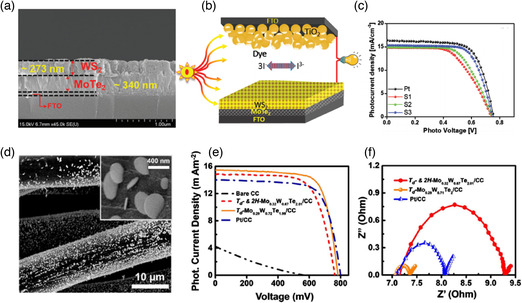
Phase‐engineered 2D TMDs based DSSC devices. a) Cross‐sectional SEM images of CE heterostructures. b) Schematic representation of the DSSC device using a WS_2_/MoTe_2_ CE. c) *J—V* curves of all electrodes including Pt at 100 mW cm^−2^ light intensity. Three thicknesses of the WS_*2*_ layer were prepared as ≈119, ≈203, and ≈273 nm, and the samples were denoted as S1, S2, and S3, respectively. The thickness of MoTe_2_ film was fixed at ≈350 nm. a–c) Reproduced with permission.^[^ ^93^ ^]^ Copyright 2018, Royal Society of Chemistry. d) SEM images of Mo_*x*_W_1–*x*_Te_2_ nanoflakes grown on CC. e) Electrochemical impedance spectroscopy for all CE. f) *J—V* curves for all CE at 100 mW cm^−2^ solar illumination. d–f) Reproduced with permission.^[^ ^49^ ^]^ Copyright 2019, Wiley‐VCH.

As the WS_2_ layer is in direct contact with the electrolyte, it plays an important role in the total catalytic activity of the composite. Therefore, the thickness of the WS_2_ thin film was varied to analyze its influence on the CE performance, while the thickness of the 1T′ MoTe_2_ layer has been fixed (**Figure** **16**a). The schematic diagram of the DSSC device is shown in Figure 16b and the curves of photocurrent density versus photovoltage (*J–V*) for all the electrodes are shown in Figure 16c in which Pt has also been analyzed for comparison. The power conversion efficiency (PCE) of the CEs was calculated as 8.5% and 7.99% for Pt and the WS_2_–MoTe_2_ electrode, respectively. This PCE is higher than the one reported for a CE solely based on sputtering‐CVD grown 1T′ MoTe_2_ (7.25%).^[^ ^95^ ^]^ The high efficiency of the double‐layer electrode could be attributed to three main factors: 1) the optimal WS_2_ film thickness (≈273 nm) that allows the proper diffusion of the electrolyte, 2) the fast charge exchange at the electrode–electrolyte interface and high number of active sites provided by MoTe_2_ due to its metallic nature, and 3) the good catalytic activity of WS_2_ produced by its sulfur‐terminated edges exposed toward the electrolyte.^[^ 96, 97 ^]^ Therefore, a synergistic effect is produced by the two materials.

Metastable alloys have also been proposed as CE for DSSC devices, Weyl semimetallic Mo_*x*_W_1–*x*_Te_2_, in particular. This material was grown as nanoflakes on carbon cloth (CC) and it was phase‐engineered by CVD to perform as a high‐performance electrocatalyst for DSSCs (Figure 16d).^[^ ^49^ ^]^ The topological Weyl semimetals (TWS) Mo_*x*_W_1–*x*_Te_2_ samples present Fermi arc surface states between Weyl nodes of opposite chirality. This unique feature enables the material to transfer charges faster than ordinary semiconductors.^[^ ^49^ ^]^ Two samples were prepared, one exhibiting a mix of T_d_ and 2H phases (T_d_‐&2H‐Mo_0.32_W_0.67_Te_2.01_) and a TWS T_d_‐Mo_0.29_W_0.72_Te_1.99_. The single‐phase CE presented the highest peak current density (*J*_PC_  = −2.72 mA cm^−2^) value which could be a result of fast charge exchange promoted by its high carrier density and mobility. On the contrary, the semiconducting phase in the T_d_‐&2H‐Mo_0.32_W_0.67_Te_2.01_ electrode reduces its catalytic performance; this electrode presented the highest charge‐transfer resistance (Figure 16e). It is also worth noting that *J*_PC_ values of T_d_ and T_d_‐&2H electrodes are higher than that of Pt/CC. This can be explained by the vertically aligned arrangement of the TMDs nanosheets which provides high number of sites to charge exchange, contrasting to the Pt/CC electrode in which the CC surface area regulates the catalytic area of the electrode. From the *J–V* curves (Figure 16f), it was found that T_d_‐Mo_0.29_W_0.72_Te_1.99_/CC presents a PCE value (8.85%) higher than those of T_d_‐&2H‐Mo_0.32_W_0.67_Te_2.01_/CC (7.81%) and even Pt/CC (8.01%) making TMD‐based TWS a great candidate for DSSCs components.

### 5.3 Photodetectors and Photovoltaic Devices

By converting light into electrical signals, photodetectors have become an important component in a great variety of devices such as image sensors, optical communications, biomedical imaging, and so on. Due to the tunable bandgaps by varying the number of layers, composition, and phase, TMDs with tailored bandgaps have been versatilely used in photodetectors presenting good photoresponse in a range of wavelengths.^[^ ^98^ ^]^ In a pioneer work of phase engineering in optoelectronic devices, Yamaguchi et al. have shown that a local 2H‐to‐1T phase transformation underneath the metal electrodes in a monolayer MoS_2_ optoelectronic device significantly reduces the native Schottky barrier and enhances the photoresponsivity.^[^ ^6^ ^]^ Apart from reducing contact resistance, the metallic or semimetallic phases of TMDs have vanishing or small bandgaps, which are suitable for the photodetection in the near‐infrared region.^[^ 39, 99 ^]^ However, due to the metastable nature of most metallic and semimetallic phases, it has been difficult to obtain these phases with high quality for making devices. CVD‐based phase engineering can therefore provide a possible solution.

**Figure 17:**
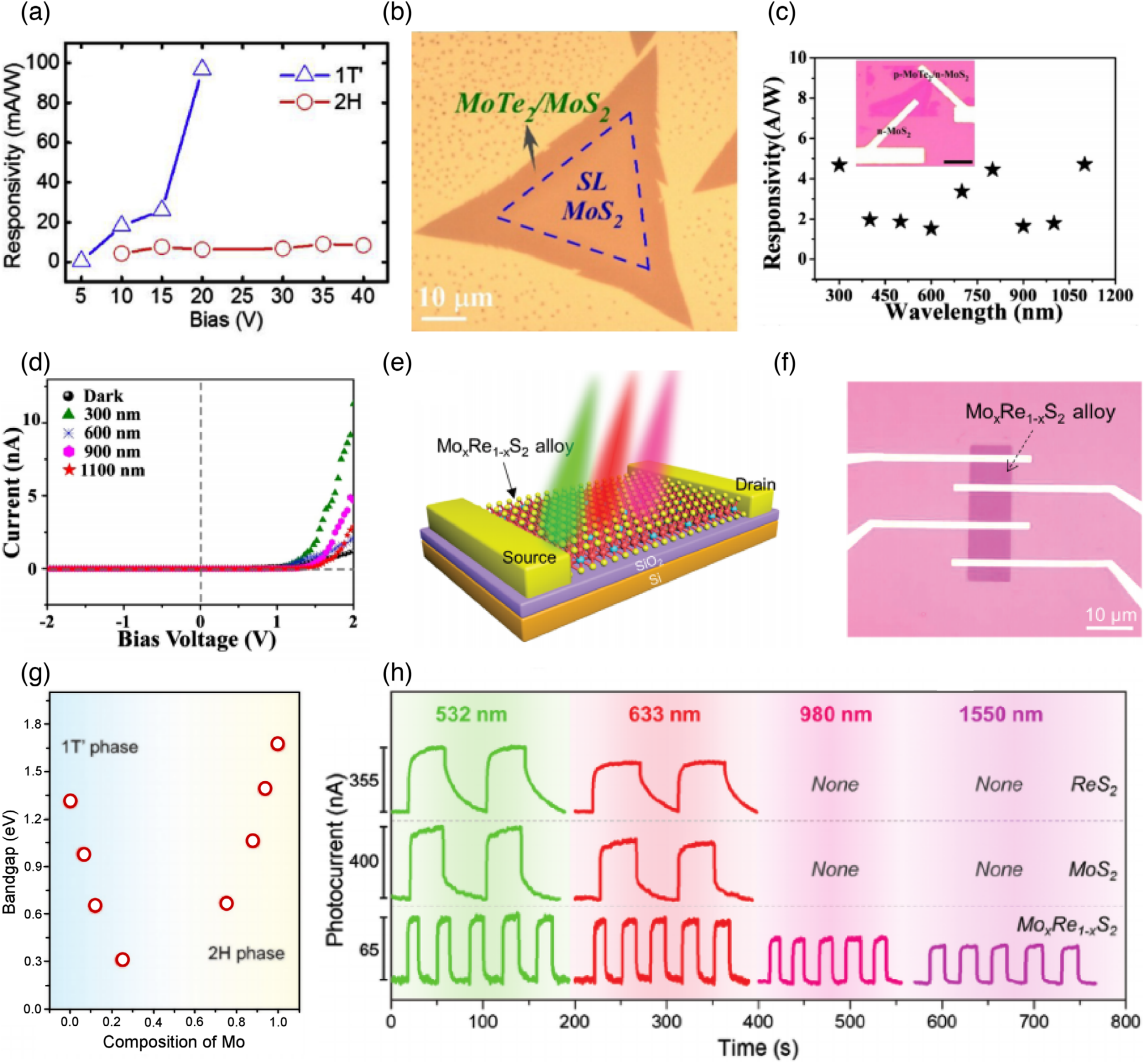
Phase‐engineered 2D TMD‐based photodetector and photovoltaic devices. a) Source—drain bias voltage versus responsivity of 1T′ and 2H MoTe_2_ photodetectors. Reproduced with permission.^[^ ^100^ ^]^ Copyright 2019, Elsevier B.V. b) Optimal image of the MoTe_2_/MoS_2_ heterostructures. c) Responsivity of the MoTe_2_/MoS_2_ heterostructures at different incident light from 300 to 1000 nm. d) *I—V* curves of the heterostructures illuminated with different wavelength lasers and in the dark. b–d) Reproduced with permission.^[^ ^101^ ^]^ Copyright 2018, Elsevier B.V. e) Schematic representation of the Mo_*x*_Re_1–*x*_S_2_ device. f) Optical microscope image of Mo_*x*_Re_1–*x*_S_2_ device. g) Bandgap as a function of the Mo composition (*x*). h) Photocurrent as a function of time for ReS_2_, MoS_2,_ and Mo_*x*_Re_1–*x*_S_2_ under light illumination with different wavelengths. e–h) Reproduced with permission.^[^ ^103^ ^]^ Copyright 2020, Wiley‐VCH.

The performance of CVD‐grown metastable 1T′ MoTe_2_ in near‐infrared photodetectors has been demonstrated.^[^ ^100^ ^]^ Under 1000 nm wavelength light illumination, the 1T′ MoTe_2_ photodetector exhibited a significantly higher photoresponsivity compared with its 2H counterpart, as shown in **Figure** **17**a, for a range of voltage biases, as explained by the higher photoconductive gain in the near‐infrared region due to the small bandgap of 1T′ MoTe_2_.

Photovoltaic devices have also been constructed with TMD bilayer heterostructures, for example, the vertical epitaxial MoTe_2_ monolayer on top of a 2H‐MoS_2_ monolayer (Figure 17b).^[^ ^101^ ^]^ The photovoltaic device was fabricated by contacting one of the two Cr/Au electrodes to the 2H MoTe_2_ and the other to the MoTe_2_/MoS_2_ bilayer region (inset in Figure 17c) and the highest responsivity (4.71 A W^−1^) was reached under 1100 nm laser illumination (Figure 17c). From the photoinduced current–voltage (*I*–*V*) curves in Figure 17d, a photovoltaic p–n junction behavior was identified at all laser wavelengths. Moreover, as the fast charge transfer between layers is essential to achieving high‐performance devices, heterostructures grown and phase‐engineered by CVD show advantages in producing clean interfaces which facilitates the interlayer charge transfer.

In addition to conventional methods for TMDs phase control, alloying is also a promising platform to modify their band structure,^[^ ^102^ ^]^ and CVD may provide an opportunity for the applications of TMD alloys in optoelectronic devices. Recently, the implementation of controlled CVD synthesis of Mo_*x*_Re_1–*x*_S_2_ alloys with tailored phases has been reported with applications in optoelectronic devices.^[^ ^103^ ^]^ Two compositions were obtained, Mo_0.98_Re_0.02_S_2_ presenting the 2H phase and Mo_0.37_Re_0.63_S_2_ exhibiting 1T′ phase. The optoelectronic properties of the alloys were analyzed by fabricating a Mo_*x*_Re_1–*x*_S_2_ alloy FET (Figure 17e,f). The effect of the alloy composition and structural phase on the bandgap is shown in Figure 17g. When the Mo concentration (*x*) increases from 0.75 to 1 in the 2H phase alloy, the bandgap changes from 0.62 to 1.67 eV, while in the 1T′ phase alloy, as *x* increases from 0 to 0.25 the bandgap presents a substantial drop from 1.31 to 0.3 eV. It is important to address the fact that this behavior is different from other 2D alloys. This is because the two different structural phases promote drastic changes in the electronic band. The photoresponse of MoS_2_, ReS_2_ (included for comparison), and Mo_*x*_Re_1–*x*_S_2_ alloy was calculated under light irradiation with different wavelengths from visible light to the near‐infrared light, as shown in Figure 17h. MoS_2_ and ReS_2_ presented high response in the visible light range but no activity was detected in the near‐infrared light, while the Mo_*x*_Re_1–*x*_S_2_ alloy exhibited response in the visible light as well as in the near‐infrared; this behavior can be explained by the bandgap. By tailoring its bandgap, the material can operate at different wavelengths.^[^ ^104^ ^]^ With these findings, the optoelectronic properties of 2D alloys may promote their implementation in photoelectronic devices.

### 5.4 HER Electrocatalysts

The electrolysis of water has become an environmentally friendly method to produce H_2_. Widely known as hydrogen evolution reaction (HER, 2H^+^ + 2e^−^ → H_2_), it is the cathodic half‐cell reaction when using typically an acid electrolyte. There are two mechanisms in which the reaction can occur, both with the same initial step which is the reduction of protons on an active site on the surface of the electrocatalyst, known as the Volmer step.^[^ ^105^ ^]^ Then, in the Volmer–Tafel mechanism two of the absorbed protons combine forming gaseous hydrogen (Tafel step). In second mechanism, Volmer–Heyrovsky, the absorbed hydrogen atom shows a tendency to combine with a proton in the acid media and an electron from the external circuit producing H_2_.^[^ ^106^ ^]^ The HER is kinetically limited by one step called rate‐limiting step. A good catalyst with suitable surface properties must ensure an efficient hydrogen‐to‐surface bond which should be neither strong nor weak.^[^ ^107^ ^]^ Several materials have been studied as electrocatalyst for HER, including metal such as platinum, metal oxides, and metallic dichalcogenides.^[^ ^108^ ^]^ TMDs have become recurrent alternative materials for research in this kind of energy applications as electrocatalysts. Particularly, MoS_2_ has been extensively studied as HER catalyst material. DFT calculations support that its basal plane is inert for hydrogen evolution, while edge sites and sulfur vacancies are catalytically active.^[^ ^109^ ^]^

It has also been reported that the 1T and related phases of TMDs have a higher number of active sites than the semiconducting 2H phase.^[^ ^110^ ^]^ This fact provides excellent opportunities to phase engineer TMD materials to improve performance in specific energy applications. The 1T phase of TMDs such as MoS_2_ and WS_2_ has been found to exhibit an outstanding performance toward the HER.^[^ 7, 111 ^]^ Thus, CVD phase engineering represents an outstanding method to produce high‐quality TMDs crystal with specific properties to further increase the activity of the electrocatalyst.

Uniform and large‐size thin 1T′ MoS_2_ has been studied as a promising electrocatalyst for HER.^[^ ^42^ ^]^ By controlling the atmosphere in the CVD process, 1T′ and 2H phases were obtained. The 1T′ phase is estimated to present a higher performance than the 2H counterpart due to the previously reported higher number of actives sites (basal plane catalytically active) and efficient electron transport..^[^ ^112^ ^]^ As predicted, 1T′ phase presented a Tafel slope of 51 mV per decade, which is lower than the 70 mV per decade shown by the 2H MoS_2_. The improved HER activity of CVD‐grown 1T′ MoS_2_ showed its potential applications as a high‐performance catalyst.

Similarly, metallic TMDs such as NbS_2_ have been studied as efficient electrocatalysts for HER. 3R and 2H phases of a nonlayered 3D polytype with an excess of niobium denoted as Nb_1+*x*_S_2_ were prepared by CVD.^[^ ^113^ ^]^ The study reveals that NbS_2_ phase is determined by the thickness of films. The performances of 2H MoS_2_, 1T MoS_2_, 1T WS_2_, 2H Nb_1.35_S_2_, 3R Nb_1+*x*_S_2_, 2H NbS_2_, and 3R NbS_2_ were measured; from the polarization curves it can be established that 2H Nb_1.35_S_2_ presents a great performance reaching 1000 mA cm^−2^ at −370 mV. In contrast, 3R Nb_1+*x*_S_2_ requires −390 mV to reach the same current density. EIS analyses were conducted on 2H Nb_1.35_S_2_ and 3R Nb_1+*x*_S_2_; the results showed that 2H Nb_1.35_S_2_ presented a series resistance of 3.5 Ω, indicating good contact with the electrode. Conversely, the 3R Nb_1+*x*_S_2_ sample showed higher resistance which agrees with observations exhibiting the 3R phase as less conducting at thicker films. Then, as the improvement of the electrocatalytic activity of TMDs is greatly depending on high conductivity, further implementation of metallic and semimetallic TMDs may guarantee efficient electrocatalytic performance.

Other considerations need to be contemplated when preparing electrocatalysts for HER. The nature of the material is an important issue but also the electrode preparation into the electrochemical cell requires careful examination. The use of polymer binders to fix the active materials into an electrode may increase the contact resistance affecting the conductivity of the material and in consequence limiting its electrocatalytic performance.^[^ ^114^ ^]^ Thus, preparing binder‐free electrocatalyst grown directly on a current collector is highly desirable. In this context, carbon cloth (CC) and 3D metallic substrates such as Ni foam have been used as substrates for the deposition of TMDs with energy applications.^[^ ^115^ ^]^ These materials are not only good current collectors but also their 3D structure improves the specific surface area and number of exposed active sites.^[^ ^116^ ^]^ Since it has been predicted that the active sites of MoS_2_ originate in Mo‐edge atoms, high density of active sites should be expected when MoS_2_ is grown on 3D substrates with large curvature gradients.^[^ 109, 117 ^]^ For this purpose, the CVD technique ensures the vapor supply and deposition of the 2D material along all the 3D structure.

**Figure 18:**
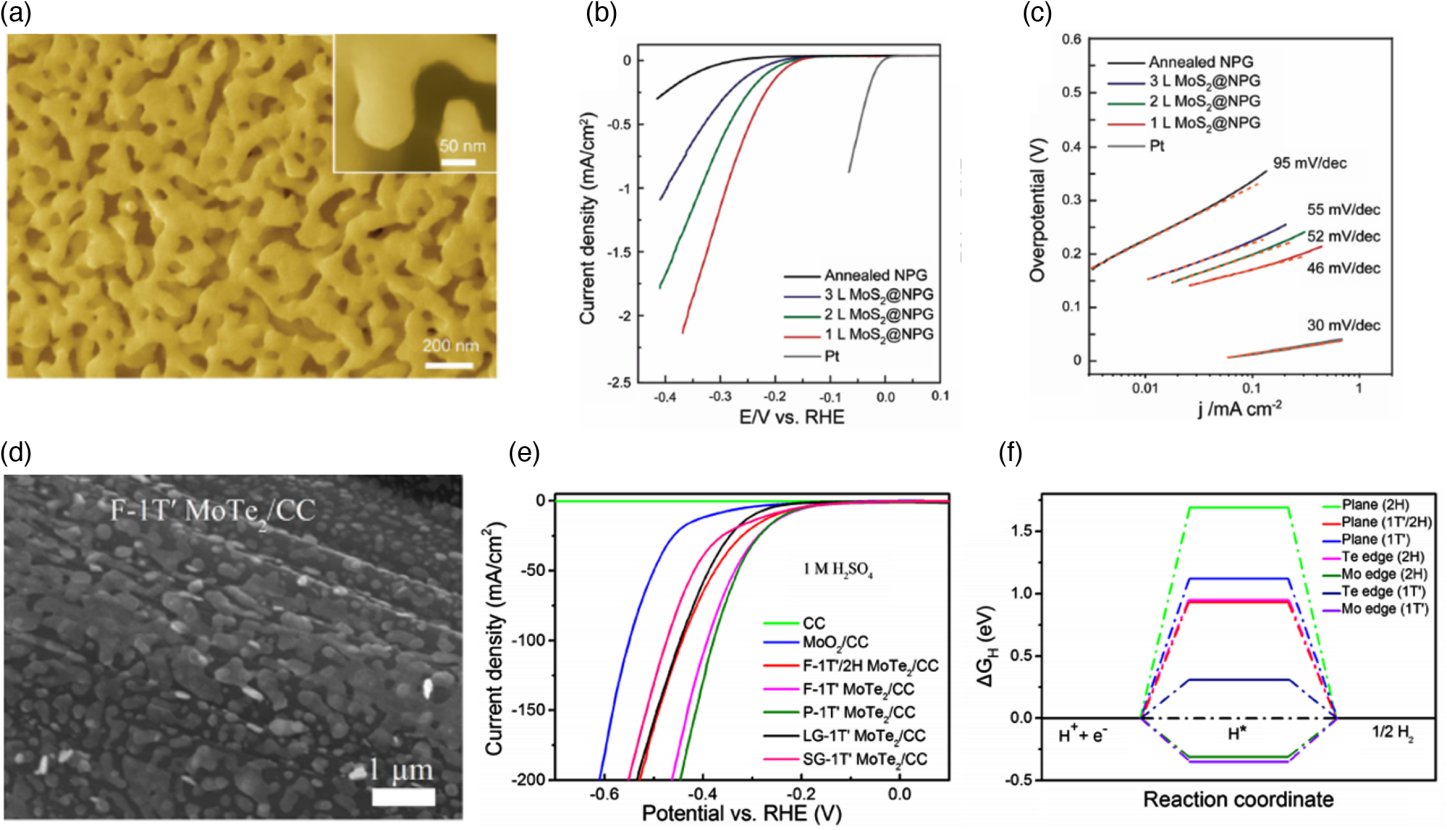
Phase‐engineered 2D TMD‐based HER electrocatalysts. a) SEM image of the MoS_2_@NPG material. b) Polarization curves of platinum, NPG, and MoS_2_@NPG samples. c) Corresponding Tafel slopes. a–c) Reproduced with permission.^[^ ^118^ ^]^ Copyright 2014, Wiley‐VCH. d) SEM image of the film‐like 1T′ MoTe_2_ deposited on CC. e) Polarization curves corresponding to all electrocatalyst where the letters F, P, LG, and SG refer to film‐like, porous, large granular, and small granular morphologies, respectively. f) The Gibbs free energies of H‐adsorption of different MoTe_2_ phases. d–f) Reproduced with permission.^[^ ^94^ ^]^ Copyright 2019, American Chemical Society.

Tan et al. deposited uniform MoS_2_ film on a 3D porous Au substrate by the CVD technique (**Figure** **18**a) and studied its electrocatalytic properties.^[^ ^118^ ^]^ The material represented lattice strains produced by the curves in the 3D substrate, providing lattice distortions into the structure. It was found that when the angle of the S—Mo—S bond is higher than 140 °C, the bandgap is close to zero and the material experiences a slightly phase transition from semiconducting to metallic phase. MoS_2_ films with different thickness were deposited and the number of MoS_2_ layers was control by adjusting the distance between the substrate and the precursor. The best HER performance was achieved by the monolayer sample presenting an overpotential of 226 mV at 10 mA cm^−2^ and a Tafel slope of 43 mV dec^−1^ (Figure 18b,c).

Recently, 1T′ MoTe_2_ has been grown on CC by the CVD method for electrocatalytic applications (Figure 18d).^[^ ^94^ ^]^ By varying the deposition temperature and Te concentration during the CVD reaction, film‐like, porous, and large and small granular morphologies were deposited and their HER activity was analyzed. As shown in Figure 18e, where the polarization curves of the different electrocatalysts are presented included a semiconducting phase and a mixture between 2H and 1T′ phases; it was found film‐like 1T′ MoTe_2_ performed better in longer periods of testing. Moreover, it was determined that the formation of defects during the deposition of the film‐like 1T′ MoTe_2_ led to a better performance.^[^ 97, 119 ^]^ The hydrogen evolution activity of the film‐like 1T′ MoTe_2_ (in 1 m H_2_SO_4_ electrolyte) showed a Tafel slope of 127.1 mV dec^−1^ and good long‐term stability. By computational calculations, the Gibbs free energy of different phases at different H‐adsorption sites have been calculated and presented in Figure 18f. By this analysis, it was determined that the edges of 2H MoTe_2_ and 1T′ MoTe_2_ present better performance than the basal planes, being 1T′ MoTe_2_ the one that exhibited the best activity. This work can inspire research in more metastable TMDs grown on 3D substrate for energy applications.

Although phase‐engineered TMDs have been shown to perform well in electronic device applications, most reports on this topic do not use CVD‐based techniques for phase engineering. For instance, magnetism can be introduced via the phase transformation because the ferromagnetic behavior has been observed in nonmagnetic 2H MoS_2_ after the transformation to the 1T phase.^[^ ^120^ ^]^ In addition, unique memristive behavior has been found to be present in MoS_2_ nanosheets containing 1T phase, leading to potential memristor device applications.^[^ ^121^ ^]^ These particular characteristics provided by the combination of phases are calling for novel technologies to implement phase engineering of TMDs in combination with versatile CVD processes. Therefore, it would be of great interest and importance to realize the aforementioned applications using CVD‐based phase engineering techniques.

## 6 Outlook and Projections

The phase‐engineered synthesis of 2D TMDs can be realized by controlling a variety of parameters involved in the CVD process. As introduced in this review, a great number of studies to date have demonstrated the CVD‐based phase engineering through approaches such as precursor control, catalytic effect introduction, reducing atmosphere control, temperature control, composition/defect control, and strain control. These CVD strategies provide direct synthesis of metastable phases without the assistance of postgrowth treatments such as alkaline intercalation, laser irradiation, or Ar plasma, which may cause structural damage to the materials.

The control of these parameters can also be combined with the features of different CVD setups. One‐step and two‐step CVD setups can be used to control the aforementioned parameters in different manners, giving flexibility in the phase engineering techniques. With the versatile CVD strategies, CVD‐based phase engineering of TMDs not only provides high‐quality single‐ or multiple‐phased materials but also facilitates the research on the properties and applications of polymorphs. Furthermore, the CVD phase‐engineered TMDs have been applied in electronics/optoelectronics and energy harvesting devices, such as FETs, photovoltaic cells, photodetectors, and HER electrocatalysts, where one specific phase or the combination of phases plays an important role.

Although numerous reports have focused on exploring the ways of obtaining desired phases and many achievements have been obtained, many fundamental and technical questions about CVD‐based phase engineering remain elusive. For example, what exactly is the mechanism for catalyst‐induced phase transformation in a CVD process, how to improve the yield of metastable phase toward 100% by CVD‐based phase engineering, and what is the best way to perform patterned phase growth in a CVD process? It is also necessary to address some key issues that need to be considered in the CVD of TMDs itself. For example, even though wafer‐scale CVD production of TMDs has been successfully achieved,^[^ ^122^ ^]^ reproducible and uniform mass production of TMDs in a controllable system remains a great challenge.

As laser irradiation has been shown to induce phase transitions,^[^ ^63^ ^]^ it is worth considering incorporating laser technology into CVD growth, for example, using the well‐known laser CVD (LCVD) to achieve phase engineering patterning growth for high‐quality TMD materials and devices. Previous reports have shed light on the great potential of using LCVD for synthesizing and pattering 2D materials, such as graphene.^[^ ^123^ ^]^ With motorized stage controlling the substrate motion, complicated patterning required by device applications can be achieved. To the best of our knowledge, LCVD‐based TMD growth and phase engineering have not been reported. Therefore, there exists plenty of room to implement and improve this approach for phase‐engineered TMD growth with unprecedented controllability.

The CVD‐based phase engineering of TMDs has been primarily focused on MoTe_2_, MoS_2,_ and several alloys. Therefore, it is important to thoroughly understand the phase transformation mechanism involved in a CVD process: thermodynamics and kinetics, and extend them to other TMD materials. Modeling and simulation of the reaction system and process are of great help to solving this problem. Moreover, the as‐grown materials in metastable phases face the risk of reverse transformation to their stable phase. Therefore, postgrowth treatment to maintain these metastable phases is essential and raises important issues in ensuring the material properties required in critical applications.

In addition to well‐established phase engineering techniques, the exploration of hybrid methods to achieve phase control on TMDs has been reported with significant results. For instance, postgrowth treatments such as in‐plane epitaxy growth have demonstrated that by filling the Te vacancies in a metallic MoTe_2_ film, phase transition can be achieved.^[^ ^124^ ^]^ It is necessary to provide further development of such techniques because the energy difference between semiconducting and metallic phases of other TMDs is higher than that of MoTe_2_, which makes the implementation of these methods in other materials a difficult task. Developing of phase engineering strategies could provide solid ground to stabilize metastable phases of other TMDs.

Finally, T_d_ phase MoTe_2_, WTe_2,_ and their alloys are Weyl semimetals.^[^ ^125^ ^]^ Facile preparation of high‐quality thin film samples of this particular phase is of great significance in exploring the novel physics of topological quantum materials. Furthermore, CVD‐based phase engineering techniques could enable novel junctions or heterostructures of different electronic phases, offering important opportunities for the development of topological quantum devices.

## Conflict of Interest

The authors declare no conflict of interest.
